# The Effect of the Creatine Analogue Beta-guanidinopropionic Acid on Energy Metabolism: A Systematic Review

**DOI:** 10.1371/journal.pone.0052879

**Published:** 2013-01-09

**Authors:** Inge Oudman, Joseph F. Clark, Lizzy M. Brewster

**Affiliations:** 1 Department of Vascular Medicine, Academic Medical Center, Amsterdam, The Netherlands; 2 Department of Neurology, University of Cincinnati, Cincinnati, Ohio, United States of America; 3 Department of Internal Medicine, Academic Medical Center, Amsterdam, The Netherlands; Université Joseph Fourier, France

## Abstract

**Background:**

Creatine kinase plays a key role in cellular energy transport. The enzyme transfers high-energy phosphoryl groups from mitochondria to subcellular sites of ATP hydrolysis, where it buffers ADP concentration by catalyzing the reversible transfer of the high-energy phosphate moiety (P) between creatine and ADP. Cellular creatine uptake is competitively inhibited by beta-guanidinopropionic acid. This substance is marked as safe for human use, but the effects are unclear. Therefore, we systematically reviewed the effect of beta-guanidinopropionic acid on energy metabolism and function of tissues with high energy demands.

**Methods:**

We performed a systematic review and searched the electronic databases Pubmed, EMBASE, the Cochrane Library, and LILACS from their inception through March 2011. Furthermore, we searched the internet and explored references from textbooks and reviews.

**Results:**

After applying the inclusion criteria, we retrieved 131 publications, mainly considering the effect of chronic oral administration of beta-guanidinopropionic acid (0.5 to 3.5%) on skeletal muscle, the cardiovascular system, and brain tissue in animals. Beta-guanidinopropionic acid decreased intracellular creatine and phosphocreatine in all tissues studied. In skeletal muscle, this effect induced a shift from glycolytic to oxidative metabolism, increased cellular glucose uptake and increased fatigue tolerance. In heart tissue this shift to mitochondrial metabolism was less pronounced. Myocardial contractility was modestly reduced, including a decreased ventricular developed pressure, albeit with unchanged cardiac output. In brain tissue adaptations in energy metabolism resulted in enhanced ATP stability and survival during hypoxia.

**Conclusion:**

Chronic beta-guanidinopropionic acid increases fatigue tolerance of skeletal muscle and survival during ischaemia in animal studies, with modestly reduced myocardial contractility. Because it is marked as safe for human use, there is a need for human data.

## Introduction

Creatine kinase (CK) is the central regulatory enzyme of cellular energy metabolism. The enzyme catalyses the reversible transfer of a phosphoryl group from ATP to creatine, creating ADP and phosphocreatine. The enzyme is mainly expressed in tissues with high energy demands, including skeletal muscle, heart, and brain. In the mitochondrial intramembraneous space, octameric CK facilitates the formation of phosphocreatine, which is transported to subcellular locations of ATP consumption. Here, ATP is rapidly regenerated in situ by cytosolic, dimeric CK (isoenzymes CK-MM, CK-MB, CK-BB). The CK-system thus functions as an effective cellular energy buffer and transport system, coupling mitochondrial ATP production to cytosolic ATP utilization, with an ATP regenerating capacity greater than from oxidative phosphorylation and glycolysis together [1]–[6].

Despite the central role in energy metabolism, CK-knockout mice, deficient in either mitochondrial or cytosolic CK, are viable. Furthermore, CK-M deficient hindlimb muscles of mice show 2-fold higher aerobic energy generating potential, lack burst activity, but exhibit improved endurance, leading to a shift from type II to type I fiber predominance [7]–[9].

Similar physiological alterations may be induced by beta-guanidinopropionic acid (βGPA or N-(aminoiminomethyl)-beta-alanine), an amino acid with a similar molecular structure as creatine, that reduces the flux through the CK reaction, by reducing cellular creatine uptake [10]–[12]. βGPA is phosphorylated in cytoplasm, but both βGPA and phosphorylated βGPA are “inefficient substrates” for the CK reaction: in vitro Vmax values are <1% of the Vmax values of creatine and phosphocreatine [13], [14]. Furthermore, in contrast to creatine, βGPA is not utilised by mitochondrial CK.15 Thus, βGPA may modulate function of tissues with high energy demands. This amino acid can be freely obtained for human use. However, to our knowledge, the effects and potential side effects of βGPA are unclear, with some reports suggesting heart failure like complications with chronic use [16]. Therefore, we conducted a systematic review to assess the effect of βGPA on energy metabolism.

## Methods

### Outcomes

The primary outcome was the effect of ßGPA on energy metabolism, function, and morphology of tissues with high and fluctuating energy demands.

### Search strategy

We sought to identify all publications, published or unpublished, that considered the effect of ßGPA on tissues with high energy demands in humans and animals. To identify relevant publications, we searched MEDLINE, EMBASE, the Cochrane Controlled Trials Register, and LILACS from their inception through March 2011. We also searched the Database of Abstracts of Reviews of Effects, Best Evidence, and Reviews in Progress (all from the United Kingdom). Finally, we searched for studies by using references from textbooks, narrative reviews, and systematic reviews; by contacting experts; and by searching the internet. We applied no language restriction.

### Selection criteria

We included all controlled studies that considered the effect of ßGPA in vivo or in vitro, in animal models or humans.

### Data assessment

At least 2 reviewers independently assessed each eligible study. Disagreement was resolved through final discussion. Our primary outcome measure was the percentage difference in means. In addition, we assessed the weighted difference in means, using the random effects model. We assessed studies for heterogeneity in baseline animal characteristics, intervention (dose and duration), analytical methods, and expression of the different outcomes and decided whether we should aggregate studies.

We aggregated studies when the direction of the outcome was homogeneous. Furthermore, we used I^2^ statistics to quantify the proportion of total variation in the estimates of the effect of intervention that was due to statistical heterogeneity and explored the sources of the heterogeneity [17]. When the I^2^ value is >75%, the variability in the effect estimates due to statistical heterogeneity is considered high, and the pooled results are less reliable [18]. We performed sensitivity analyses by reanalyzing data using fixed-effects models. Studies without numerical data were described. We predefined subgroups of different animal species, age (at baseline), muscle type, and dose and duration intervention. Cut off points for age (at baseline), baseline weight (animal, skeletal muscle, and heart), and duration of intervention were determined post hoc based on available data. Statistical analyses were performed with the Cochrane Review Manager (RevMan) software, version 5 (Cochrane Collaboration, Oxford, United Kingdom) and with the SPSS statistical software package for Windows (Microsoft, Redmond, Wash), version 19.0 (SPSS inc, Chicago, III).

## Results

### Study retrieval

**Figure 1:**
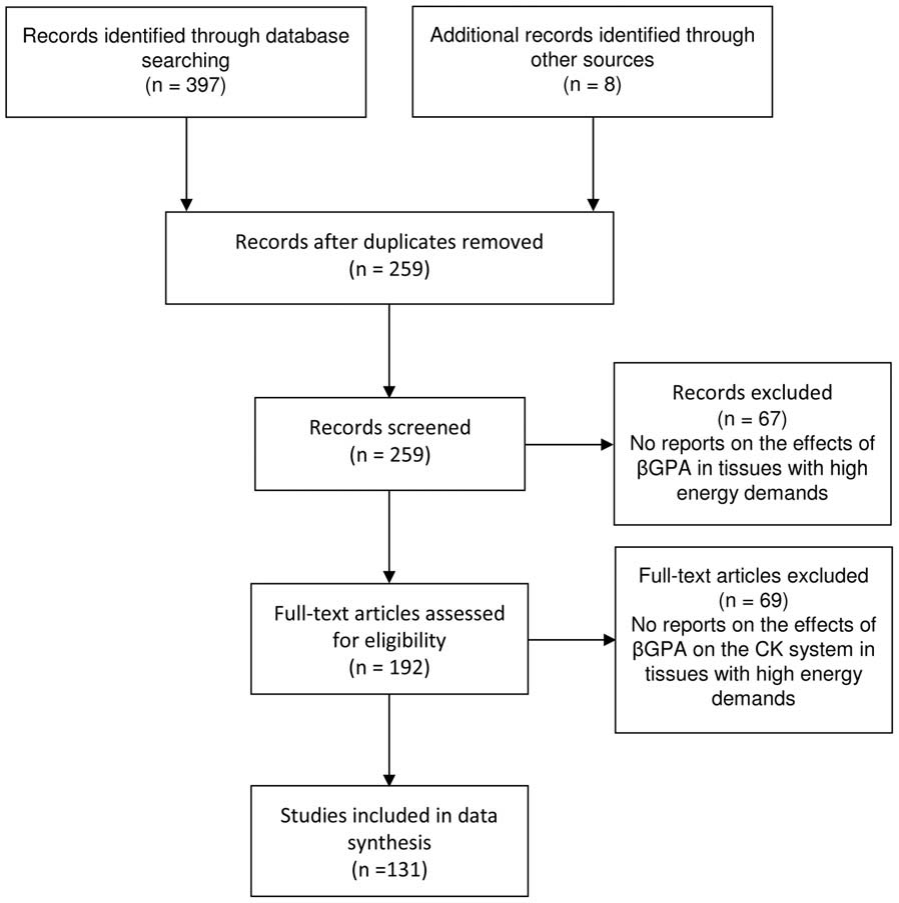
Flow diagram. Flow chart of reports retrieved in databases. CK, creatine kinase; βGPA, beta-guanidinopropionic acid.

Full reports or abstracts from 259 references yielded 131 eligible papers in animals (n = 120) and humans (n = 11) (Figure 1: Study Flow Chart).

### Methodological quality of included studies

None of the included studies reported randomization. Blinding or dropouts were not described. Studies used preset doses of βGPA and did not report whether they assessed the minimum dose needed to reach a maximum effect, although a dose-response curve was assessed in one study [19]. All animal studies included a control group.

### Heterogeneity analysis

Studies were heterogeneous regarding species (rats, mice, guinea pigs, turkey poults, frogs, and rhesus monkeys), sex, skeletal muscle fiber type predominance (slow “type I” or fast “type II”), part of the heart (total heart, left, or right ventricle), βGPA dose, duration of intervention, and outcome measures. Because of the small numbers in other species, only studies in rodents were pooled.

### Body weight and food intake

**Figure 2:**
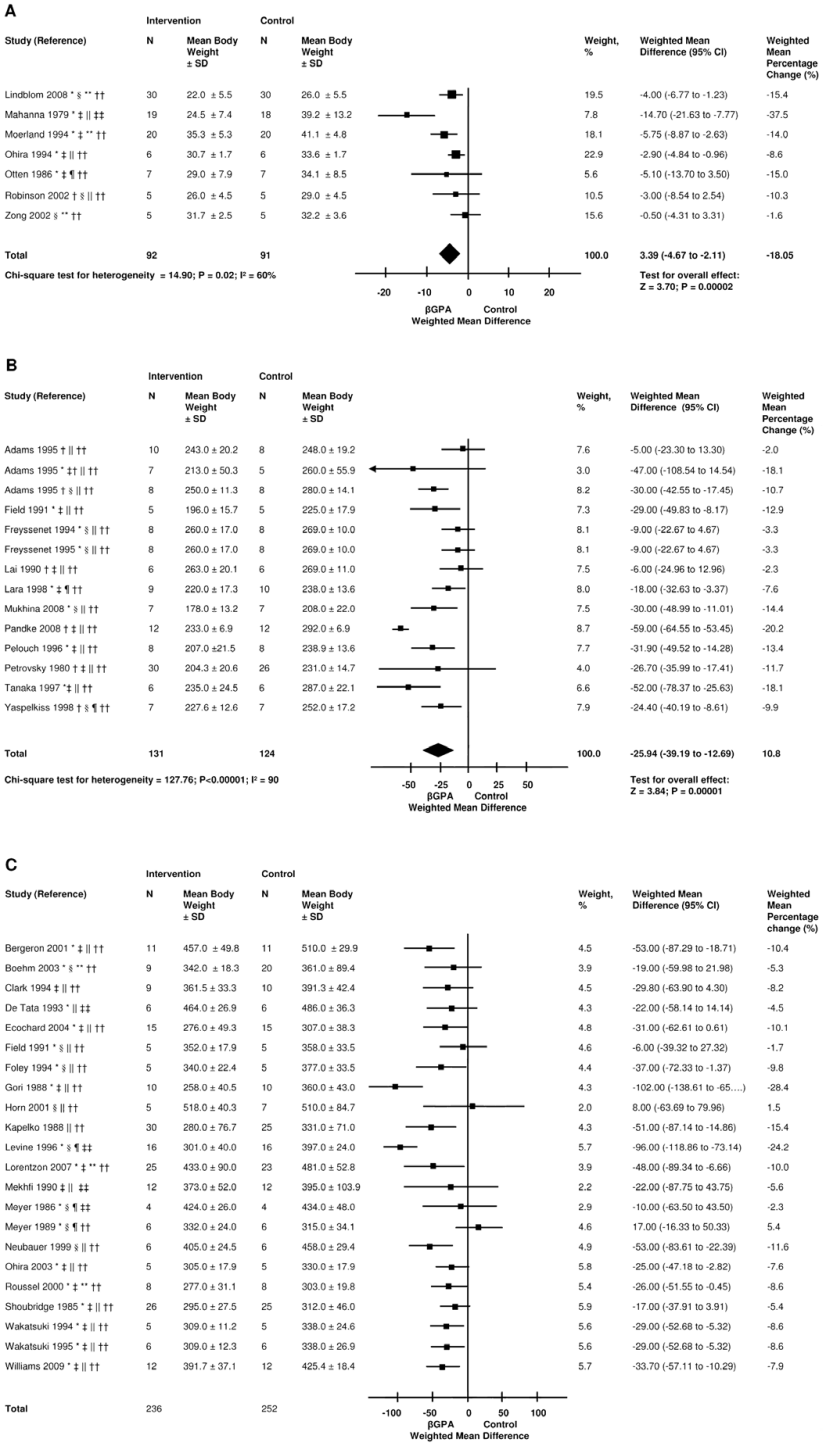
The effect of βGPA on body weight. The effect of βGPA on body weight of mice with a baseline body weight less than 50 grams (A), rats with a baseline bodyweight less than 300 grams (B) and more than or equal to 300 grams (C). This is a random-effects model. Squares are weighted mean differences in body weight. The size of the squares represents study weight and horizontal lines represent 95% CIs. Arrowheads depict data outside the scale. Black diamonds are pooled estimates. The weighted mean percentage change was calculated from the percentage change per study and the sample size. Results for the effect of βGPA on body weight of rats with a baseline body weight of more than 300 grams are not pooled because of heterogeneity in the direction of effect. * Male sex; † Female sex; ‡ Animal age ≤6 weeks; § Animal age >6 weeks; ∥ Dose of βGPA = 1% in the diet or drinking water; ¶ Dose of βGPA = 2% in the diet or drinking water; ** Dose of βGPA = other; †† Duration of intervention ≤10 weeks; ‡‡ Duration of intervention >10 weeks.

Of 117 papers in animals, 41 papers reported the effect of βGPA on body weight [16], [20]–[59]. Studies with weight outcomes included rats (median age 32, range 15 to 84) and mice (median age 77, range 21 to 210). The dose of βGPA ranged from 0.8 to 2.5%. The median duration of intervention was 56 days (range, 4 to 240). These studies showed an average weight decrease of 10.1% (SD 7.6) (Figure 2). In five studies with numerical information on ad libitum food intake, there was no evidence of reduced food intake (data not shown) [12], [20], [35], [56], [60]. One study proposed weight loss due a central hypophagic effect, showing that ßGPA (single intrathecal injection) resulted in a 9-fold increased Fos expression, a measure of hypothalamus activity, in rats [61].

#### Subgroup analyses

We performed subgroup analyses for species (i.e. mouse vs. rat), baseline weight (post hoc cut off based on available data: <300 grams, ≥300 g), and duration of intervention (post hoc cut off: (≤3 weeks; 3 to 10 weeks; >10 weeks), and found similar magnitudes and directions of the point estimate in these subgroups (data not shown).

### Skeletal muscle

Studies included rats (median age 33, range 21 to 84) or mice (median age 56, range 21 to 84). The dose of βGPA ranged from 1 to 2.5% (diet or intraperitoneal injection). The median duration of intervention was 56 days (range 14 to 240).

#### Creatine, phosphocreatine, and ATP

**Figure 3:**
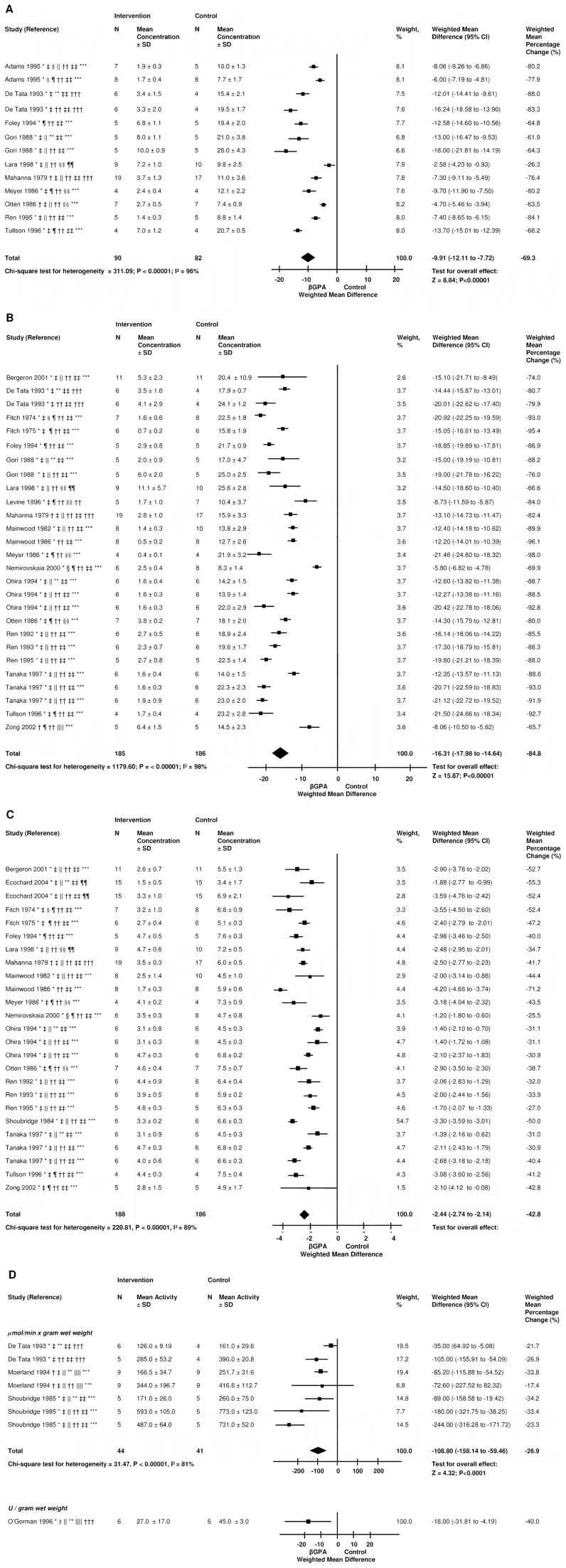
The effect of βGPA on the creatine kinase system of skeletal muscle. The effect of βGPA on skeletal muscle creatine (A), phosphocreatine (B), and ATP (C) in µmol per gram dry weight, total creatine (creatine+phosphocreatine) and mitochondrial creatine kinase activity (D), hexokinase (E),. This is a random-effects model. Squares are weighted mean differences in body weight. The size of the squares represents study weight and horizontal lines represent 95% CIs. Arrowheads depict data outside the scale. Black diamonds are pooled estimates. The weighted mean percentage change was calculated from the percentage change per study and the sample size. * Rat; † Mouse; ‡ Male sex; § Female sex; ∥ Animal age ≤6 weeks; ¶ Animal age >6 weeks; ** Type I fiber predominant muscle; †† Type II fiber predominant muscle; ‡‡ Dose of βGPA = 1% in the diet or drinking water; §§ dose of βGPA = 2% in the diet or drinking water; ∥∥ Dose of βGPA = other; ¶¶ Duration of intervention ≤3 weeks; *** Duration of intervention 3 to 10 weeks; ††† Duration of intervention >10 weeks.

Included studies showed decreased creatine, phosphocreatine, total creatine (creatine+phosphocreatine), and ATP levels of respectively 66.1% (SD 19.2), 79.7% (SD 21.6), 86.7% (SD 10.0), and 38.8% (SD 13.6) after ßGPA (Figure 3 and Figure S1) [11], [12], [20], [22], [25], [31]–[33], [36], [37], [40], [43], [44], [48]–[51], [53], [54], [56], [57], [62]–[73]. We excluded one study with conflicting results (unchanged creatine, as well as a 56% increase) [71].

#### CK activity, reaction velocity and flux

Total muscle CK activity decreased by 28.6% (SD 7.2) (Figure 3) [26], [36], [54], [74]. In contrast to total muscle CK activity, mitochondrial CK activity was unchanged, and densitometry analysis showed a 3-fold increase of mitochondrial CK (Figure S1) [26], [74].

#### Adenylate kinase and AMP-deaminase

One study reported a 165% (no SD available) increase in skeletal muscle adenylate kinase activity after βGPA,74 while AMP-deaminase activity was 70.1% (SD 19.1) reduced (data not shown) [67], [72], [75].

#### Glycolysis

Glycolytic enzyme activities were reported to decrease after βGPA, including phosphorylase by 38.8% (SD 17.7), and lactate dehydrogenase by 16.2% (SD 10.6) [26], [34], [54], [68]. Phosphofructokinase and α-glycerophosphate dehydrogenase showed non-significant changes in activity of resp. 4.0 (SD 70.4) and 21.4% (SD 32.6) (data not shown) [26], [54], [59], [68].

#### Mitochondria

Mitochondrial oxidative enzymes were reported to increase after βGPA, including citrate synthase (+20.7%, SD 18.5), succinate dehydrogenase (+71.2%, SD 44.5), hydroxyacyl-Coa dehydrogenase (+38.6%, SD 27.5), 2-oxoglutarate dehydrogenase (+130%, no SD available), and hexokinase (+23.8%, SD 34.5), whereas cytochrome oxidase increased by 23.2% (SD 10.4) in type II fiber predominant muscle and decreased by 9.1% (SD 5.7) in type I fiber predominant muscle (Figure S1B) [22], [26], [31], [36], [43], [44], [49], [50], [53], [54], [59], [68], [74], [76]. Other mitochondrial markers reported to increase after βGPA were cytochrome c protein level by 41.2% and adenine nucleotide transporter level by 133.8% (no SDs available) (data not shown) [68], [74].

#### Other enzyme systems

AMP-activated protein kinase (AMPK) protein levels were reported to increase in 6 studies [20], [22], [31], [56], [77], [78], of which two provided a percentage change [31], [77]. These studies showed a 45% increased AMPK protein content and 20% increased AMPK mRNA in mice [77], but no difference in rats after βGPA [31].

#### Glucose uptake and insulin resistance

In general, studies reported a 64% (SD 55.0) increased skeletal muscle glucose uptake (data not shown) [19], [33]. In line with this, GLUT-4 protein content, a sarcolemmal insulin responsive glucose transporter, increased in 4 studies of which one provided a percentage increase; 45% in type I and 33% in type II fiber predominant muscle [21], [31], [68], [79]. Consequently, studies showed an 94.3% (SD 43.5) increased skeletal muscle glycogen content after βGPA (Figure S1) [25], [26], [33], [37], [68], [80]. Results for plasma glucose were more pronounced in diabetic than non-diabetic animals; fasting glucose levels were decreased by resp. 54.5 (SD 31.4) and 5.1% (SD 10.4) (Figure S1) [20], [31], [33], [59], [66]. Insulin levels followed a similar trend, with decreases in fasting plasma insulin of resp. 78.9 (no SD available) and 26.6% (SD 20.9) (Figure S1) [19], [20], [31], [33].

#### Cellular fatty acid metabolism

One study reported that fatty acid transporter protein content increased by resp. 21.0% and 55.0% in type I and type II fiber predominant muscle of rats and plasma free fatty acid concentration by 66.7% after βGPA [31]. In contrast, 2 other studies reported unchanged fasting plasma concentrations of fatty acids, triglycerides, and total lipids in rats, but numerical details were not provided [30], [56].

#### Muscle function: In vivo endurance capacity

Endurance capacity was increased during normal workload in rats (age 21 days), as measured by swimming to exhaustion with 2.5% of body weight attached or in a treadmill run, whereas endurance capacity at higher workload (swimming to exhaustion with 5.0% of body weight attached) was significantly reduced, without providing numerical details [25], [34].

#### *In situ* muscle function

When assessed under general anaesthesia in situ, resistance to fatigue was reported to improve after ßGPA in 7 studies [14], [23], [24], [29], [37], [72], [81], of which two provided numerical details, including a 166.3% (SD 27.9) increased maximal length of time to maintain isometric muscle contraction in type I fiber predominant muscle, which was not significantly changed in type II fiber predominant muscle (−9.8% (SD 16.4) [14], [29], and a 54.8% increased residual force in type II fiber predominant muscle after a fatiguing protocol (data not shown) [37]. Initial isometric peak force was not significantly decreased by 4.2% (SD 16.6) after ßGPA (Figure S1) [29], [37], [72], [81]. In line with this, 6 studies reported a reduced potentiation of force after ßGPA in type I and II fiber predominant muscle of rats, without providing numerical details [11], [37], [38], [65], [72], [81].

#### Isolated muscle function

Isolated muscle peak twitch force and peak tetanic force showed no significant change after ßGPA; an average change of resp. +0.19% (SD 35.6) and −7.7% (SD 27.5) (Figure S1) [23], [24], [36], [43], [51], [62], [64], [82]–[84]. With regard to muscle contraction, the maximum rate of tension development decreased by 27.0% (SD 21.8), time to reach maximal contraction rate increased by 70.8% (SD 75.4), and contraction time showed no significant changes (+9.8% (SD 9.7) (data not shown) [23], [24], [36], [43], [62], [82]–[84]. In line with this, a 43.8% (SD 14.3) decreased maximum rate of relaxation, an 80.8% (SD 57.7) increased time to reach maximum relaxation rate, and a 13.9% (SD 17.9) increased one-half relaxation time was reported (data not shown) [23], [24], [36], [43], [62], [82]–[84]. Fatigue resistance of type II fiber predominant muscle, indicated by residual force in after train stimulation, was reported to increase in two studies, but five reported no significant difference, without providing details [23], [24], [36], [62], [82]–[84].

#### Morphology

In general, studies reported a shift from type IIb fibers to type IIa and I fibers in type II fiber predominant muscle and a shift from type IIa to type I fibers in type I fiber predominant muscle (data not shown) [34], [43], [51], [53], [57], [66], [69], [76], [81], with a decrease in absolute and relative muscle weight of resp. 18.6 (SD 8.9) and 10.2% (SD 10.1) (Figure S1) [23]–[25], [28], [29], [34], [36], [43], [45], [48]–[50], [53], [54], [57], [59], [76], [82]. This apparent shift towards oxidative metabolism was accompanied by a 2-fold increase in mitochondrial DNA [20], and increased mitochondrial density [56], [78], with occasional subsarcolemmal accumulation of enlarged, elongated, or irregular shaped mitochondria, containing paracristalline inclusions [48], [54], [74], [81], [85], [86]. The paracrystalline inclusions, in some studies, were found to be condensed and crystallized mitochondrial CK [54], [86].

#### Sensitivity analyses

For all outcomes, reanalyzing the data by using fixed-effects models, resulted in similar magnitudes of effect and conclusions about statistical heterogeneity, although the confidence intervals were narrower, as expected.

#### Subgroup analyses

We performed subgroup analyses for species (rats and mice), sex, muscle type (type I and II fiber predominant muscle, ßGPA dose, duration of intervention (≤3 weeks; 3 to 10 weeks; >10 weeks), and found similar magnitudes and directions of the point estimate in all study groups (data not shown).

### Heart

Studies included rats, mice, and guinea pigs (median age 51 days, range 15 to 82 days), with ßGPA 0.5 to 2% in the diet or through an osmotic minipump (1 M, 2.5 µL/hour). The median duration of intervention was 56 days (range 20 to 116 days).

#### Creatine, phosphocreatine, and ATP

**Figure 4:**
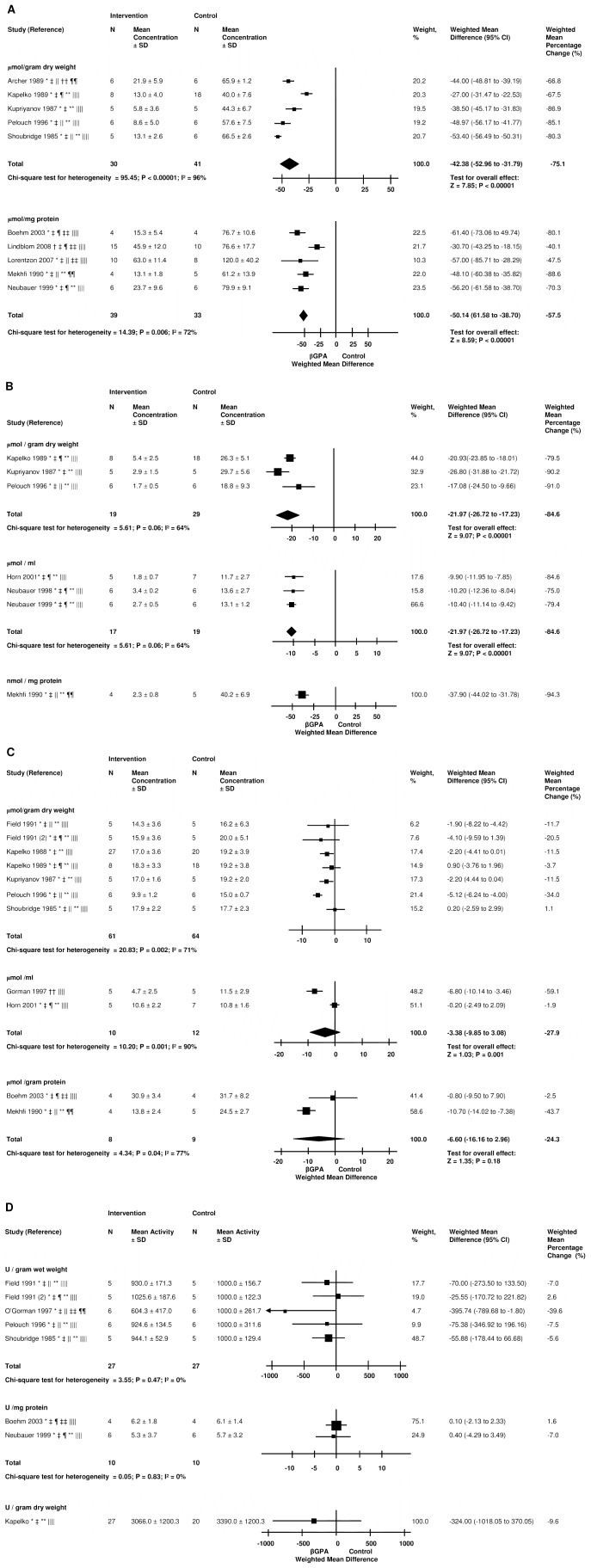
The effect of βGPA on the creatine kinase system of the heart. The effect of βGPA on myocardial total creatine (A), phosphocreatine (B), ATP, and creatine kinase activity (D). This is a random-effects model. Squares are weighted mean differences in body weight. The size of the squares represents study weight and horizontal lines represent 95% CIs. Arrowheads depict data outside the scale. Black diamonds are pooled estimates. The weighted mean percentage change was calculated from the percentage change per study and the sample size. * Rat; † Mouse; ‡ Male sex; § Female sex; ∥ Animal age ≤6 weeks; ¶ Animal age >6 weeks; ** Dose of βGPA = 1% in the diet or drinking water; †† Dose of βGPA = 2% in the diet or drinking water; ‡‡ Dose of βGPA = other; §§ Duration of intervention ≤3 weeks; ∥∥ Duration of intervention 3 to 10 weeks; ¶¶ Duration of intervention >10 weeks.

As in skeletal muscle, myocardial creatine, phosphocreatine, and total creatine decreased after ßGPA; an average reduction of respectively 62.4% (SD 19.3), 82.5% (SD 8.3), and 68.9% (SD 17.2) (Figure 4) [30], [35], [39], [41], [42], [47], [55], [87]–[94]. Myocardial ATP concentration decreased by 17.8% (SD 18.6) in vivo (Figure 4) and 30 to 40% (no SD available) in vitro [30], [39], [46], [47], [52], [55], [87], [88], [90], [92], [94], [95].

#### CK activity, reaction velocity, and flux

Total myocardial CK activity was not significantly changed (10.1% (SD 14.0)) after βGPA (Figure 4) [30], [35], [46], [52], [55], [74], [90]. In line with this, the cytoplasmatic isoforms CK-MM, CK-MB, and CK-BB remained unchanged in vivo, but changed in vitro by resp. −31.6 (p<0.05), −58.2 (p<0.05), and +26.3% (p>0.05) after incubation of cardiomyocytes [30], [52], [55], [89]. In addition, mitochondrial CK decreased by 16.2% (p>0.05) (Figure S2) [30], [52], [55], [74]. The flux through CK-reaction and the CK reaction velocity decreased by resp. 67.0% (SD 23.7) and 46.9% (SD 11.0) after ßGPA [96]–[101].

#### Adenylate kinase and glycolysis

In contrast to skeletal muscle, myocardial activities of adenylate kinase and the glycolytic enzymes phosphofructokinase, phosphorylase, and lactate dehydrogenase were not significantly changed after ßGPA by resp. +17.6, −8.7, 0.0, and −8.1% (SDs not available) (data not shown) [74], [90].

#### Mitochondria

Myocardial oxidative enzymes including citrate synthase, 2-oxoglutarate dehydrogenase, 3-hydroxyacyl-Coa dehydrogenase, hexokinase, succinate dehydrogenase, and cytochrome c oxidase activity were not significantly changed (resp. −6.6 (SD 5.8), +5.1, +1.0, −29.2, −33.0, and −3.7%, SDs not available) (Figure S2) [26], [52], [74], [102].

#### *In vivo* cardiac function

During baseline performance ßGPA resulted in unchanged left ventricular systolic pressure, cardiac output, and rate of tension development, with a 69.1% reduced left ventricular end-diastolic pressure [35], but two other studies reported a 37.7% (SD18.7) reduced left ventricle fractional shortening, an echocardiographic measure of left ventricular function [41], [42]. During high workload, studies showed unchanged peak left ventricular developed pressure and cardiac output during aortic occlusion,35 a 15.7% reduced left ventricular shortening during increased pacing [41], and a reduced peak left ventricular blood pressure and heart rate (no numerical details) (Figure S2) [58]. Mortality rates after the induction of myocardial infarction were increased: 93.5 to 100% after ßGPA vs. 0 to 46.6% in controls [41], [47], [91].

#### Function of perfused hearts and isolated cardiac fibers

Despite an average left ventricular developed pressure reduction of 24.9% (SD 17.2) and a 39.7% (SD 10.1) reduction in tension development after ßGPA, cardiac output was unchanged (105.2% (SD 10.5), with unchanged heart rate and coronary flow [35], [39], [46], [90], [91], [95], [103]. Left ventricular end-diastolic pressure was 81.3% increased (Figure S1) [46]. Myocardial oxygen consumption was increased by 18.7% in the right ventricle [30], but unchanged in the left ventricle [90], [95]. At high workload, left and right ventricular pressure were resp. reduced by 14.3% (no SD available) and unchanged, with unchanged left ventricular cardiac output [46], [90]. In line with this, ßGPA had no effect on resting tension, maximum tension, and tension at increasing calcium concentrations of isolated myocardial fiber bundles, although the time to reach original tension after a quick stretch was 37.3% increased, indicating a slowing of the cross-bridge turnover [39]. No effect on maximal force, rate of tension development, and half relaxation time of isolated papillary muscles was reported [93].

#### Morphology

As in skeletal muscle, studies reported a shift from fast to slow myosin isoforms, including a 22.3% (SD 13.6) decrease of fast isoforms with a 3.8-fold (SD 3.1) increase of slow isoforms [39], [58]. Wet heart weight, heart weight to body weight ratio (HW/BW), wet left ventricle weight, and left ventricle to body weight ratio were not significantly increased after ßGPA by resp. 7.3 (SD 9.6), 11.8 (SD 14.6%), 27.4 (SD 27.8), and 19.4% (SD 18.8) [16], [30], [35], [39], [41], [42], [47], [55], [58], [91], while one study reported a 25.4% decreased dry heart weight (Figure S2) [46]. Mitochondrial density was unchanged after ßGPA without visible inclusions [74], [103], but perturbations in mitochondrial form and DNA were reported in two other studies [102], [104], including cylindrically shaped mitochondria with paracrystalline inclusions with condensed and crystallized Mi-CK after in vitro incubation with ßGPA [104], and a 76% increased proliferation of left ventricle mitochondrial organelles with 22% increased mitochondrial DNA (densitometric values) [102].

#### Sensitivity analyses

When we reanalyzed the data by using fixed-effects models, we found similar magnitudes of effect and conclusions about statistical heterogeneity.

#### Subgroup analyses

Subgroup analyses for animal species, sex, animal age, dose and duration of intervention (≤10 weeks; >10 weeks, defined post hoc based on available data) all showed similar magnitudes and directions of the point estimate (data not shown).

### Vascular smooth muscle

#### The creatine kinase reaction

No papers reported data on vascular creatine concentration. Regarding phosphocreatine, acute perfusion of porcine carotid arteries with ßGPA 50 mM during 12 hours, resulted in a 19.4 to 22.4% decreased phosphocreatine concentration [105]. In addition, phosphocreatine was 86% reduced in the rat portal vein after ßGPA 2% during 63 days [106]. Perfusion of porcine carotid arteries with ßGPA resulted in a 55.4% decreased ATP concentration with glucose in the perfusion solution, compared to 32.4% with pyruvate in the solution [105]. In the rat portal vein, ATP concentration was unchanged [106].

### Vascular function

Spontaneous contractile activity or developed force of the rat portal vein was unchanged after ßGPA [106]. In pulmonary vascular smooth muscle, ßGPA 2% during 4 months to rats (aged 56 days) had no significant effect on normoxic pulmonary arterial pressure, maximum rise in pressure during hypoxia, or peak pulmonary arterial pressure in response to angiotensin 2, a pulmonary vasoconstrictor [107].

### Brain and nervous system

#### Creatine, phosphocreatine, and ATP

Brain creatine, phosphocreatine, and ATP levels decreased by resp. 25.9% (SD 3.0), 26.9% (SD 10.8), and 25% (no SD available) after ßGPA [70], [108], [109]. This was reported in total brain, cortex and striatum of mice and rats (median age 28 days, range 21 to 112 days) after ßGPA 1 to 2.5% in the diet for 6 weeks or after daily intraperitoneal injection (0.2 ml of 0.5 M) for 12 weeks [70], [108], [109]. In vitro, creatine concentration in hippocampal slices of mice was reduced by 50.0% after ßGPA (10 mM for 3 hours) [110]. In line with this, a 67% inhibition of creatine uptake was reported in rat telencephalum after incubation with ßGPA (500 µM), showing that creatine transporter inhibition is also present in brain [111]. However, in vivo, the rate of phosphocreatine decrease in brain was reported to be slower than in skeletal muscle (2% of baseline concentration per week vs 5 to 10% decrease per week in skeletal muscle) [109].

#### CK activity, reaction velocity and flux

In stark contrast with skeletal muscle and heart, CK activity of total brain and white matter were increased by resp. 77.2 (SD 28.4, p<0.05) and 50.0% (SD not available, p<0.05) after ßGPA (2 to 3.5%) for 6 weeks to 4 months, but CK activity of grey matter was unchanged (−11.3%, no SD available, p>0.05) [74], [112], [113]. Regarding mitochondrial CK activity, an increase of 53.7% (no SD available, p<0.05) was found after ßGPA in one study [74], with no change in mitochondrial CK immunoreactivity reported in one other study [114]. However, CK reaction velocity and flux were resp. 65 and 75% reduced after ßGPA 2 to 2.5% during 42 to 70 days [112], [113].

#### Adenylate kinase

AK activity of total brain and white matter were resp. increased by 105.9% (no SD available, p<0.05) and decreased by 16.7% (no SD available, p<0.05). In grey matter AK was increased by 42.9% (no SD available, p<0.05) [74], [112].

#### Mitochondria

A 2-fold increased activity of succinate dehydrogenase, was reported in 1 study in rats (21 days) after ßGPA 3.5% [74]. Furthermore, mRNA of mitochondrial markers was increased, including cytochrome C in cortex and striatum (resp. 120 and 145%, p<0.05), cytochrome oxidase IV in the cortex (120%, p<0.05), with unchanged activity in striatum (+120%, p>0.05), and cytochrome oxidase II in striatum (150%, p<0.05), with unchanged activity in the cortex (−2.5%, p>0.05) [108].

#### Other enzyme systems

AMPK activity was increased by 20% (no SD available) in the cortex and 42.5% (SD 24.7) in striatum of mice after ßGPA [60], [108].

#### Functional effects: Seizures

During seizures induced with pentylenetetrazole, no significant changes in brain ATP were observed in either ßGPA-fed mice (2% for 21 days) or controls (no numerical details). Moreover, in brain of ßGPA-fed mice phosphocreatine increased during seizures compared to baseline (no numerical details), while in brain of control mice phosphocreatine decreased by 10 to 20%.113 In line with this, during seizures the flux through the CK reaction increased by 40% from baseline in the ßGPA-fed mice, while in controls induction of seizures did not change the CK reaction flux [113]. Thus, ATP stability was not impaired during a high energy demanding seizure state. Regarding seizure activity, one study reported spontaneous epileptiform discharges in rats (age 63 days) after ßGPA (daily i.p. injections with 400 mg/kg during 7 days), but ßGPA had no effect on seizure activity in a rat model of chronic epilepsy [114].

#### Functional effects: Hypoxia

During and after hypoxia, no reductions in brain phosphocreatine and ATP compared to baseline were reported among ßGPA-fed mice, while hypoxia resulted in phosphocreatine and ATP reductions of 25 to 30% in controls. Moreover, during hypoxia CK reaction flux increased among ßGPA-fed mice (no numerical details), while hypoxia did not change CK reaction flux in controls [113]. Importantly, ßGPA resulted in decreased mortality during and after ischaemia; 8.3% versus 38.5% in controls (p<0.05) [112]. Thus, through adaptations in CK isoenzymes and ATP metabolic pathways, ßGPA may protect the brain from ATP loss during hypoxic, and perhaps other, states of ATP deprivation.

#### Neurodegenerative diseases

In mice (aged 70 to 84 days) with Parkinson's disease and the associated mitochondrial loss of function (mimicked via injections with a mitochondrial complex-1 inhibitor) ßGPA (1%) increased neuronal mitochondrial density by 16.1% (p<0.05) and mitochondrial number per cell by 33.3% (p<0.05), with unchanged mitochondrial volume compared to a 29.1% reduction in controls [60]. Thus, ßGPA might have a protective effect on mitochondrial function.

#### Morphology

In contrast to skeletal muscle, no mitochondrial inclusion bodies were reported in brain after ßGPA 3.5% for 3 to 4 months [74], [108]. However, as in skeletal muscle, mitochondrial density in neurons of the substantia nigra was 16.1% increased in mice after ßGPA 1% for 32 days [60], and increased mitochondrial DNA content (no numerical details shown) was reported in striatum and cortex after ßGPA during 10 weeks [108]. Finally, mild proliferation of astrocytes was reported in mice (aged 16 weeks) after ßGPA during 10 weeks, indicating neural development [108].

### Kidney

Data on the kidney were scarce, with two studies reporting a reduced creatine uptake of kidney cortex brush-border membrane vesicles of fetal, newborn, and adult rats after incubation with 20 to 1000 millimolair βGPA [115], [116].

### Brown adipose tissue

Rectal and tail temperature of resting rats was reported to decrease by resp. 3.0 (SD 0.4) and 20.1% (SD not available) after βGPA [117], [118]. Brown adipose tissue weight, influenced by reduced body temperature, was reported to increase by 3.4% (SD 6.0) [117], [118].

### Tumor growth

A 63% (SD 14.1) reduced proliferation of intraperitoneal Ehrlich ascites tumor cells and a growth delay of mammary carcinoma of 1.6 days (SD 0.3) was reported after βGPA (through intraperitoneal injection, intravenous injection, or the diet) [79], [119], [120]. In addition, reduced food intake due to expansion of the tumor was prevented by βGPA (132.0% of control, no SD available) [119].

### Humans

#### Uptake of βGPA and the effect on human cell lines and plasma

Regarding intestinal absorption in humans, one study showed that βGPA was reported to have high affinity for transport by the human intestinal proton-coupled amino acid transporter (hPAT1) in vitro [121]. In addition, effects of βGPA on human cell lines were reported in 10 studies, including a 30 to 40% decrease in creatine and phosphocreatine content, without alterations in ATP after incubation of endothelial cells (5 mM βGPA for 24 hours) [122], and increased immunostaining of myoglobin, a marker for slow oxidative fibers after incubation of isolated myoblasts (20 µM βGPA for 7 days) [77].

In red blood cells, a 45% reduced creatine transport was reported after incubation with βGPA (1 mM) [123]. In these red blood cells, studies reported a reduced hexose monophosphate shunt activity, indicated by a 55.8% (SD 2.13) reduced glucose-6-phosphate dehydrogenase activity after incubation with βGPA (20 µg) [124], [125], and a 20% increased ascorbate cyanide test after incubation of healthy human plasma with βGPA (20 µM) [126]. In line with this, increased in vitro haemolysis was reported after adding high doses of βGPA (340 to 1770 µM) to plasma of healthy volunteers, without providing numerical details [127]. Regarding white blood cells, a reduced mitogenic response of lymphocytes was found in the presence of βGPA (10 mM) [128], while in neutrophils a 50% decreased ATP concentration and a 25% reduced activation was reported 3 hours after βGPA incubation (5 µM) [129], [130].

## Discussion

In this systematic review we show that ßGPA is an effective inhibitor of the flux through the CK reaction. In skeletal muscle, chronic intervention with ßGPA led to markedly reduced creatine, phosphocreatine, and ATP concentrations and reduced cytosolic CK activity, while mitochondrial CK activity was increased. This generally led to a shift from glycolytic to mitochondrial oxidative metabolism, resulting in a shift from type II to type I fiber predominance, increased glucose tolerance, and reduced skeletal muscle and body weight, comparable to alterations reported after endurance exercise [131]–[137].

The reduced body weight with unchanged food intake may be related to the fiber type shift, as an association between type II fiber predominance and body weight was previously reported [138]–[141]. In addition, cellular fatty acid transporter protein concentration and fatty acid oxidative capacity were increased after ßGPA, which may lead to reduced lipid storage [31]. However, with limited evidence for unchanged food intake, weight loss may as well be a consequence of reduced intake [12], [20], [35], [56], [60].

Several compensating mechanisms for the reduced cellular phosphagen content may lead to the observed fiber type shift. First, activation of AMPK is thought to defend against energy deficiency in skeletal muscle by stimulating mitochondrial biogenesis and alternative oxidative ATP generating pathways, with increased glucose transport and fatty acid oxidation [20], [20], [50], [56], [142]–[145]. In addition, through mitochondrial proliferation, the mean diffusion distance between mitochondria and contractile elements, the myofibrils, decreases. In this way, tissues may compensate for the attenuated spatial buffer capacity by the CK system [146]. Furthermore, the AK system may compensate for decreased CK activity, by providing a comparable energy-shuttling system. Increased cytosolic ADP is converted to AMP via cytoplasmic AK, which is used by mitochondrial AK in the intermembranous space to stimulate oxidative phosphorylation [8], [74], [147]–[149].

In line with the shift towards oxidative metabolism, the changes in skeletal muscle performance were comparable with functional changes after endurance training, i.e. increased fatigue tolerance with mildly reduced peak performance. This is in accord with studies in mice with a genomic deletion of CK, resulting in a mildly symptomatic phenotype with reduced muscle peak performance, showing that CK is relevant but not indispensible [150], [151].

In contrast to skeletal muscle, cytosolic CK activity, AK activity, and oxidative enzymes of heart tissue were unchanged after βGPA, with reduced mitochondrial CK activity. Consistent with this, βGPA was not phosphorylated by mitochondrial CK [15], [16], [74]. This differential response in cardiac and skeletal muscle was also found in rodents after endurance training, in which myocardial mitochondrial respiration chain activity and citrate synthase activity were unchanged [133], [152], [153]. As energy is mainly supplied by mitochondrial fatty acid respiration, the myocardium may have sufficient pre-existent oxidative capacity to supply the energy requirement induced by βGPA [154], [155].

As in skeletal muscle, myocardial contractile performance was minimally reduced. During baseline performance, the isolated heart ejected an equal cardiac output in spite of a slowed and reduced left ventricular developed pressure. This finding may be explained by a slower ejection rate after βGPA, which may facilitate a more prolonged and complete left ventricular ejection in spite of the reduced ventricular pressure [94]. In vivo left ventricular pressure was unchanged, suggesting that the intact rat cardiac and/or humoral compensatory mechanisms, such as catecholamines and sympathetic and vagal innervation, may be sufficient to maintain normal function [35].

In contrast with the functional effects on skeletal muscle, the myocardial effects were not comparable with the effects of endurance training. It is known that endurance training may result in eccentric myocardial hypertrophy, increased stroke volume and decreased heart rate, leading to unchanged or decreased cardiac output at rest [156], [157]. During high workload, however, the endurance trained heart is able to reach a much higher cardiac output than the untrained heart, which we did not find in these data.

Compared to the myocardium and skeletal muscle, brain concentrations of creatine, phosphocreatine and ATP were less decreased after βGPA. It was previously hypothesized that this was due to the existence of two compartments in the brain, one in which creatine is replaced by βGPA and one in which creatine is not replaced [109]. Another explanation might be that brain cells can synthesise creatine, but this is disputed since humans and mice with an absence of brain creatine transporter do not appear to have creatine in the brain [158]–[161]. Total brain CK activity was increased, mainly due to an increase in white matter. However, brain CK catalyzed reaction rates were markedly reduced, with increased mitochondrial function [60], [162]. These findings may as well be explained by brain compartmentalization. Grey matter is characterized by the presence of CK-B and Mi-CK isoenzymes, with relatively slow CK-catalyzed reaction rates, similar to smooth muscle. On the other hand, white matter, with ≈50% of the CK activity of grey matter, contains higher CK-reaction rates, and little or no Mi-CK, comparable to skeletal muscle [163]. The effects of βGPA in brain may therefore reflect inhibition of cellular creatine uptake in predominantly white matter, with the reduced CK-catalyzed flux a property of grey matter [113]. Intriguingly, βGPA protected brain tissue of mice from ATP loss during seizures and mortality during hypoxia, by yet unknown mechanisms [112], [113].

The effect of βGPA on tissue composition and function seems reversible, as almost no residual changes in enzyme activities of type I and II fiber predominant muscle were reported one month after withdrawal and no residual changes in mitochondrial morphology after four months [54]. Repletion with creatine after βGPA resulted in an even faster recovery of biochemical and functional alterations induced by βGPA [23], [24], [62].

A limitation of this review is the lack of human data, despite the over the counter availability of βGPA. Furthermore, the sample size of the included studies was often small, which resulted in considerable statistical heterogeneity for several outcomes.

In summary, reversible inhibition of the CK-system with βGPA in animal studies modulated metabolism towards enhanced mitochondrial function and oxidative capacity in skeletal muscle, with less marked alterations in heart muscle, probably due to the fact that βGPA is not utilised by mitochondrial CK in the myocardium. These modulations led to an increase in endurance capacity and insulin sensitivity of skeletal muscle, minimally altered contractility in the heart, and a greater tolerance for cerebral energy deprivation during seizures and hypoxia. The variation in metabolic and functional adaptations of the tissues studied in this review, suggest a tissue specific regulation of energy metabolism, and physiology of the CK reaction. Notably, despite the fact that βGPA can be purchased for human use, there is lack of human data.

## Supporting Information

Figure S1 **The effect of βGPA on the creatine kinase system of skeletal muscle.** The effect of βGPA on skeletal muscle creatine (A), phosphocreatine (B), and ATP (C) in µmol per gram dry weight, total creatine (µmol per gram wet weight) and mitochondrial creatine kinase activity (D), hexokinase (E), citrate synthase (F), and glycogen (E); the effect on plasma glucose (H) and insulin (I); the effect on in situ (J) and isolated (K/L) skeletal muscle function, and the effect on rat skeletal muscle weight (M). This is a random-effects model. Squares are weighted mean differences in body weight. The size of the squares represents study weight and horizontal lines represent 95% CIs. Arrowheads depict data outside the scale. Black diamonds are pooled estimates. The weighted mean percentage change was calculated from the percentage change per study and the sample size. * Rat; † Mouse; ‡ Male sex; § Female sex; ∥ Animal age ≤6 weeks; ¶ Animal age >6 weeks; ** Type I fiber predominant muscle; †† Type II fiber predominant muscle; ‡‡ Dose of βGPA = 1% in the diet or drinking water; §§ dose of βGPA = 2% in the diet or drinking water; ∥∥ Dose of βGPA = other; ¶¶ Duration of intervention ≤3 weeks; *** Duration of intervention 3 to 10 weeks; ††† Duration of intervention >10 weeks.
(PDF)

Figure S2 **The effect of βGPA on the creatine kinase system of the heart.** The effect of βGPA on myocardial total creatine (A), phosphocreatine (B), ATP, and creatine kinase activity (D). This is a random-effects model. Squares are weighted mean differences in body weight. The size of the squares represents study weight and horizontal lines represent 95% CIs. Arrowheads depict data outside the scale. Black diamonds are pooled estimates. The weighted mean percentage change was calculated from the percentage change per study and the sample size. * Rat; † Mouse; ‡ Male sex; § Female sex; ∥ Animal age ≤6 weeks; ¶ Animal age >6 weeks; ** Dose of βGPA = 1% in the diet or drinking water; †† Dose of βGPA = 2% in the diet or drinking water; ‡‡ Dose of βGPA = other; §§ Duration of intervention ≤3 weeks; ∥∥ Duration of intervention 3 to 10 weeks; ¶¶ Duration of intervention >10 weeks.
(PDF)
