# Supplementary material for: The Effect of the Creatine Analogue Beta-guanidinopropionic Acid on Energy Metabolism: A Systematic Review
Source: PLoS One. 2013 Jan 9;8(1):e52879. doi: 10.1371/journal.pone.0052879 (PMC3541392; doi:10.1371/journal.pone.0052879)
Supplement: Figure S1 — The effect of βGPA on the creatine kinase system of skeletal muscle. The effect of βGPA on skeletal muscle creatine (A), phosphocreatine (B), and ATP (C) in µmol per gram dry weight, total creatine (µmol per gram wet weight) and mitochondrial creatine kinase activity (D), hexokinase (E), citrate synthase (F), and glycogen (E); the effect on plasma glucose (H) and insulin (I); the effect on in situ (J) and isolated (K/L) skeletal muscle function, and the effect on rat skeletal muscle weight (M). This is a random-effects model. Squares are weighted mean differences in body weight. The size of the squares represents study weight and horizontal lines represent 95% CIs. Arrowheads depict data outside the scale. Black diamonds are pooled estimates. The weighted mean percentage change was calculated from the percentage change per study and the sample size. * Rat; † Mouse; ‡ Male sex; § Female sex; ∥ Animal age ≤6 weeks; ¶ Animal age >6 weeks; ** Type I fiber predominant muscle; †† Type II fiber predominant muscle; ‡‡ Dose of βGPA = 1% in the diet or drinking water; §§ dose of βGPA = 2% in the diet or drinking water; ∥∥ Dose of βGPA = other; ¶¶ Duration of intervention ≤3 weeks; *** Duration of intervention 3 to 10 weeks; ††† Duration of intervention >10 weeks. (PDF) [file pone.0052879.s001.pdf]

A

Study (Reference)	Intervention N	Intervention Mean Concentration ± SD	Control N	Control Mean Concentration ± SD	Weight, %	Weighted Mean Difference (95% CI)	Weighted Mean Percentage Change (%)
Eijnde 2004 † ‡ ¶ ** ‡‡ ¶¶	12	19.4 ± 5.5	12	30.9 ± 5.9	10.2	-11.50 (-16.07 to -6.93)	-37.2
Eijnde 2004 † ‡ ¶ †† ‡‡ ¶¶	12	23.0 ± 4.9	12	36.6 ± 7.6	10.1	-13.60 (-18.71 to -8.49)	-37.2
Freyssenet 1994 * ‡ ¶ †† ‡‡ ***	8	17.8 ± 2.7	8	36.3 ± 7.3	10.0	-18.50 (-23.89 to -13.11)	-51.0
Freyssenet 1995 * ‡ ¶ ** ‡‡ ***	7	8.0 ± 3.8	8	38.8 ± 7.2	10.0	-30.80 (-36.53 to -25.07)	-79.4
Freyssenet 1995 * ‡ ¶ †† ‡‡ ***	7	17.8 ± 2.7	8	36.2 ± 6.7	10.1	-18.40 (-23.46 to -13.34)	-50.8
Pandke 2008 * § || ** ‡‡ ***	12	9.6 ± 2.4	12	48.0 ± 9.7	10.0	-38.40 (-44.06 to -32.74)	-80.0
Shields 1973 * ‡§ || †† ‡‡ ¶¶	6	6.3 ± 0.2	5	37.7 ± 4.1	10.4	-31.41 (-35.00 to -27.82)	-83.4
Shields 1975 * ‡ || †† ‡‡ ***	6	5.9 ± 1.4	6	45.3 ± 5.0	10.3	-39.43 (-43.55 to -35.31)	-87.0
Shields 1975 * ‡ || †† ‡‡ ***	6	5.0 ± 1.8	6	37.2 ± 4.0	10.4	-32.20 (-35.67 to -28.73)	-76.6
Williams 2009 * ‡ || ** ‡‡ ***	3	8.5 ± 2.1	3	55.9 ± 18.9	5.0	-47.40 (-68.89 to -25.91)	-84.8
Williams 2009 * ‡ || †† ‡‡ ***	3	14.8 ± 1.6	3	81.3 ± 26.5	3.3	-66.50 (-96.54 to 36.46)	-81.8
**Total**	**124**		**117**		**100.0**	**-28.48 (-35.09 to -21.87)**	**-62.8**

Chi-square test for heterogeneity = 267.78; P = < 0.00001; I² = 94%

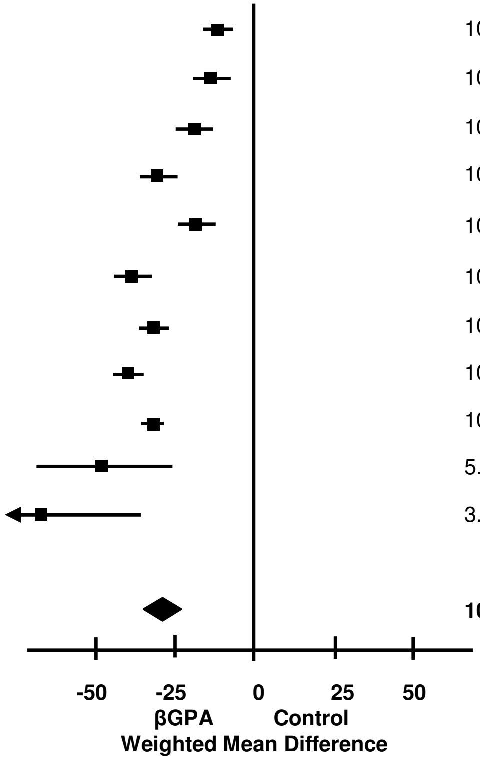

Test for overall effect:
Z = 8.45; P<0.00001

B

Study (Reference)	Intervention N	Intervention Mean Concentration ± SD	Control N	Control Mean Concentration ± SD	Weight, %	Weighted Mean Difference (95% CI)	Weighted MeanPercentage Change (%)
Eijnde 2004 † ‡ ¶ ** ‡‡ ¶¶	12	20.4 ± 6.6	12	44.5 ± 13.9	12.6	-24.10 (-32.78 to -15.42)	-16.8
Eijnde 2004 † ‡ ¶ †† ‡‡ ¶¶	12	81.1 ± 9.7	12	135.5 ± 21.1	10.9	-54.40 (-67.55 to -41.25)	-5.1
Freyssenet 1994 * ‡ ¶ †† ‡‡ ***	8	22.5 ± 3.1	8	87.9 ± 7.2	13.5	-65.40 (-70.83 to -59.97)	-26.1
Freyssenet 1995 * ‡ ¶ ** ‡‡ ***	7	7.1 ± 2.1	8	55.8 ± 5.6	13.8	-48.70 (-52.88 to -44.52)	-87.3
Freyssenet 1995 * ‡ ¶ †† ‡‡ ***	7	22.5 ± 3.1	8	88.2 ± 6.7	13.6	-65.70 (-70.88 to -60.52)	-84.5
Pandke 2008 * § || ** ‡‡ ***	12	4.1 ± 0.7	12	18.1 ± 2.0	13.9	-43.60 (-47.34 to -39.86)	-91.4
Williams 2009 * ‡ || ** ‡‡ ***	3	0.5 ± 0.4	3	42.5 ± 13.0	10.3	-42.00 (-56.70 to -27.30)	-98.8
Williams 2009 * ‡ || †† ‡‡ ***	3	4.4 ± 2.1	3	58.1 ± 10.2	11.4	-53.70 (-65.50 to -41.90)	-92.4
**Total**	**64**		**66**		**100.0**	**-49.97 (-59.05 to -40.88)**	**-52.8**

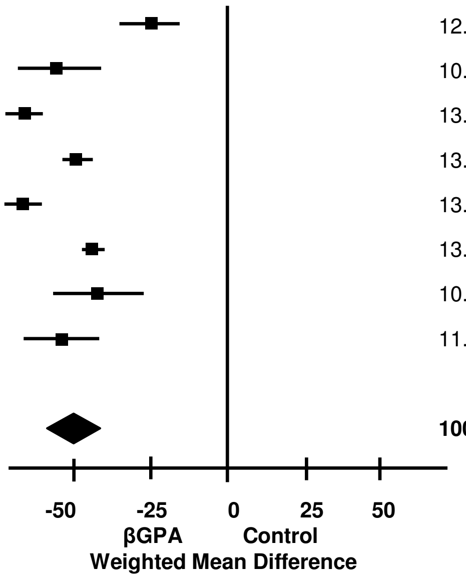

Chi-square test for heterogeneity = 112.89, P < 0.00001, I^2 = 94%

Test for overall effect:
Z = 10.78; P<0.00001

C

Study (Reference)	Intervention N	Intervention Mean Concentration ± SD	Control N	Control Mean Concentration ± SD	Weight, %	Weighted Mean Difference (95% CI)	Weighted Mean Percentage Change (%)
Eijnde 2004 † ‡ ¶ ** ‡‡ ¶¶	12	13.9 ± 2.8	12	16.7 ± 6.2	8.4	-2.80 (-6.66 to 1.06)	-16.8
Eijnde 2004 † ‡ ¶ †† ‡‡ ¶¶	12	28.2 ± 5.5	12	29.7 ± 6.2	6.4	-1.50 (-6.22 to 3.22)	-5.1
Freyssenet 1994 * ‡ ¶ †† ‡‡ ***	8	22.4 ± 0.8	8	30.2 ± 1.8	19.0	-7.80 (-9.23 to -6.37)	-25.8
Freyssenet 1995 * ‡ ¶ ** ‡‡ ***	7	15.0 ± 2.1	8	22.9 ± 1.4	16.8	-7.90 (-9.73 to -6.07)	-34.5
Freyssenet 1995 * ‡ ¶ †† ‡‡ ***	7	22.4 ± 0.8	8	30.2 ± 1.8	19.2	-7.80 (-9.18 to -6.42)	-25.8
Pandke 2008 * § || ** ‡‡ ***	12	10.6 ± 1.4	12	19.8 ± 2.8	17.3	-9.20 (-10.95 to -7.45)	-46.5
Williams 2009 * ‡ || ** ‡‡ ***	3	5.4 ± 2.3	3	17.9 ± 4.5	4.8	-12.50 (-18.19 to -6.81)	-69.8
Williams 2009 * ‡ || †† ‡‡ ***	3	2.8 ± 1.5	3	4.9 ± 1.7	8.1	-9.40 (-13.39 to -5.41)	-35.1
**Total**	**64**		**66**		**100.0**	**-7.59 (-8.99 to -6.19)**	**-27.6**

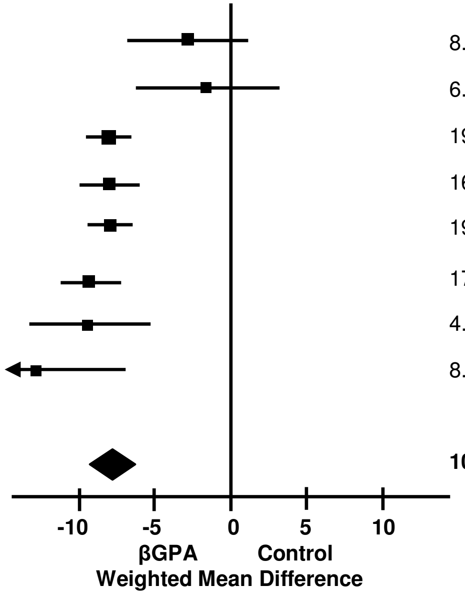

Chi-square test for heterogeneity = 18.95; P = 0.008; I^2 = 63%

Test for overall effect:
Z = 10.65; P<0.00001

D

Study (Reference)	Intervention N	Intervention Mean Concentration ± SD	Control N	Control Mean Concentration ± SD	Weight, %	Weighted Mean Difference (95% CI)	Weighted Mean Percentage Change (%)
**Total Creatine, µmol / gram wet weight**							
Fitch 1974 * ‡ § ¶ †† ‡‡ ***	7	9.6 ± 2.4	9	38.7 ± 5.1	11.8	-29.08 (-32.87 to -25.29)	-75.2
Foley 1994 * ¶ †† ‡‡ ***	5	9.7 ± 1.3	5	41.1 ± 1.4	12.7	-31.44 (-33.13 to -29.75)	-76.4
Levine 1996 * ‡ ¶ †† §§ ††	5	4.7 ± 2.0	7	21.2 ± 1.88	12.5	-16.47 (-18.67 to -14.27)	-77.8
Mainwood 1986 * †† ‡‡ ***	8	5.2 ± 0.6	8	26.7 ± 2.7	12.6	-21.50 (-23.42 to -19.58)	-80.5
Moerland 1989 † ‡ || ** |||| ***	5	1.1 ± 0.6	5	28.1 ± 2.1	12.6	-27.02 (-28.93 to -25.11)	-96.1
Moerland 1989 † ‡ || †† |||| ***	5	1.8 ± 0.6	5	39.2 ± 2.2	12.6	-37.34 (-39.37 to -35.31)	-95.4
Moerland 1994 † ‡ || ** |||| ***	5	1.1 ± 0.6	5	21.5 ± 2.4	12.5	-20.48 (-22.68 to -18.28)	-94.9
Moerland 1994 † ‡ || †† |||| ***	5	1.8 ± 0.6	5	31.8 ± 1.4	12.7	-29.95 (-31.29 to -28.61)	-97.5
**Total**	**45**		**49**		**100.0**	**-26.66 (-31.17 to -22.15)**	**-85.4**

Chi-square test for heterogeneity = 297.79, P < 0.00001, I^2 = 98%

Test for overall effect: Z = 11.58; P < 0.00001

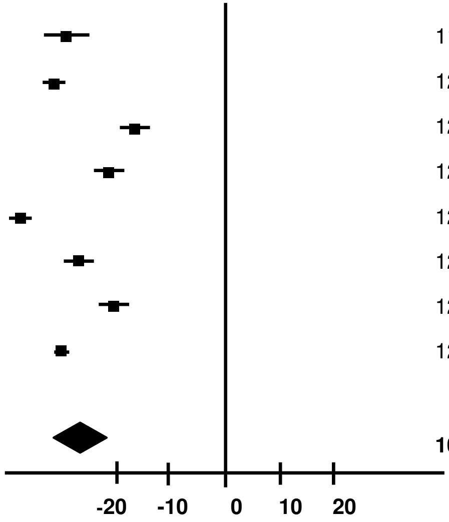

Mitochondrial creatine kinase (densitometric analysis of immuno-stained Western blots)

Study (Reference)	Intervention N	Intervention Mean Concentration ± SD	Control N	Control Mean Concentration ± SD	Weight, %	Weighted Mean Difference (95% CI)	Weighted Mean Percentage Change (%)
O'Gorman 1996 * § || ** |||| †††	3	7543.0 ± 1432	3	1544.0 ± 196.0	100.0	5999.00 (4363.46 to 7634.54)	388.5

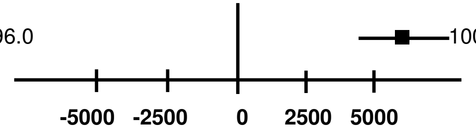

Mitochondrial creatine kinase activity (nmol/min per mg protein)

Study (Reference)	Intervention N	Intervention Mean Concentration ± SD	Control N	Control Mean Concentration ± SD	Weight, %	Weighted Mean Difference (95% CI)	Weighted Mean Percentage Change (%)
Shoubridge 1985 * ‡ || †† ‡‡ ***	5	2473.0 ± 155.0	5	2484.0 ± 233.0	100.0	-11.00 (-256.29 to 234.29)	-0.4

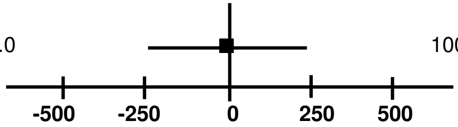

E

Study (Reference)	Intervention N	Intervention Mean Concentration ± SD	Control N	Control Mean Concentration ± SD	Weight, %	Weighted Mean Difference (95% CI)	Weighted Mean Percentage Change (%)
**µmol / min x gram wet weight**							
De Tata 1993 * ‡ ** ‡‡ †††	6	1.12 ± 0.10	4	0.87 ± 0.02	26.3	0.25 (0.17 to 0.33)	28.7
De Tata 1993 * ‡ †† ‡‡ †††	5	1.79 ± 0.43	4	1.07 ± 0.12	8.1	0.72 (0.33 to 1.11)	67.3
Freyssenet 1995 * ‡ ¶ ** ‡‡ ***	7	1.95 ± 0.44	8	1.61 ± 0.26	8.8	0.34 (-0.03 to 0.71)	21.1
Freyssenet 1995 * ‡ ¶ †† ‡‡ ***	7	1.69 ± 0.20	8	1.23 ± 0.18	18.0	0.46 (0.27 to 0.65)	37.4
O'Gorman 1996 * § || ** |||| †††	5	0.63 ± 0.40	5	1.00 ± 0.80	2.6	-0.37 (-1.15 to 0.41)	-21.2
Shoubridge 1985 * ‡ || ** ‡‡ ***	5	1.38 ± 0.22	5	1.34 ± 0.17	14.7	0.04 (-0.20 to 0.28)	3.0
Shoubridge 1985 * ‡ || †† ‡‡ ***	5	1.35 ± 0.15	5	1.05 ± 0.08	21.4	0.30 (0.15 to 0.45)	28.6
**Total**	**40**		**39**				
**Chi-square test for heterogeneity = 15.13, P = 0.02, I² = 60%**							
**µmol / min x gram protein**							
Ren 1993 * ‡ || †† ‡‡ ***	8	8.1 ± 1.8	8	5.3 ± 0.9	100.0	2.72 (1.33 to 4.11)	52.8

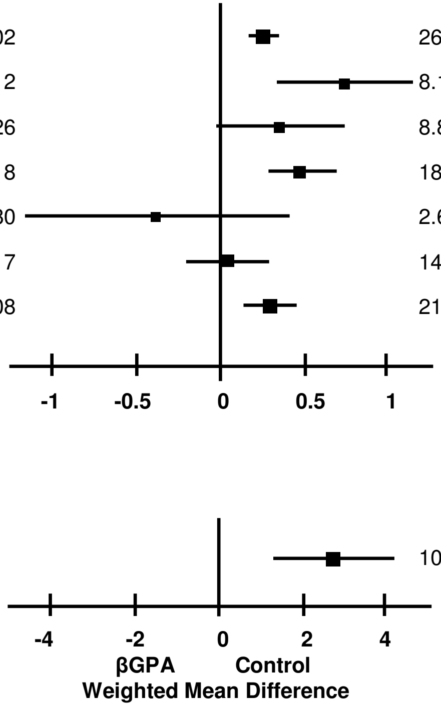

F

Study (Reference)	Intervention N	Intervention Mean Concentration ± SD	Control N	Control Mean Concentration ± SD	Weight, %	Weighted Mean Difference (95% CI)	Weighted Mean Percentage Change (%)
**μmol / min x gram wet weight**							
De Tata 1993 * ‡ †† ‡‡ †††	6	14.5 ± 5.2	6	13.8 ± 2.0	6.1	0.70 (-3.90 to 5.30)	5.1
Ecochard 2004 * ‡ || ** ‡‡ ¶¶	15	32.2 ± 13.2	15	31.0 ± 7.4	3.5	1.20 (-6.43 to 8.83)	3.9
Ecochard 2004 * ‡ || †† ‡‡ ¶¶	15	23.0 ± 4.7	15	19.9 ± 3.5	8.2	3.10 (0.16 to 6.04)	15.6
Freyssenet 1994 * ‡ ¶ †† ‡‡ ***	8	12.2 ± 2.1	8	9.3 ± 1.6	9.6	2.90 (1.07 to 4.73)	31.2
Freyssenet 1995 * ‡ ¶ ** ‡‡ ***	7	9.6 ± 1.3	8	9.8 ± 0.7	10.3	-0.20 (-1.28 to 0.88)	-2.0
Freyssenet 1994 * ‡ ¶ †† ‡‡ ***	7	12.2 ± 0.1	8	9.0 ± 1.7	9.4	3.20 (1.25 to 5.15)	35.6
Lara 1998 * ‡ || †† §§ ¶¶	10	40.7 ± 14.1	10	35.8 ± 11.7	1.8	4.90 (-6.82 to 16.62)	13.7
Moerland 1994 † ‡ || ** |||| ***	6	25.8 ± 3.4	6	25.9 ± 7.4	4.3	-0.06 (-6.55 to 6.43)	-0.2
Moerland 1994 † ‡ || †† |||| ***	6	23.3 ± 4.4	6	14.8 ± 4.4	5.7	8.46 (3.49 to 13.43)	57.1
Pandke 2008 * § || ** ‡‡ ***	12	58.2 ± 5.2	12	48.2 ± 6.6	6.0	10.00 (5.25 to 14.75)	20.7
Shoubridge 1985 * ‡ || ** ‡‡ ***	5	19.1 ± 1.7	5	19.6 ± 3.1	8.0	-0.50 (-3.60 to 2.60)	-1.0
Shoubridge 1985 * ‡ || †† ‡‡ ***	5	21.2 ± 2.2	5	15.0 ± 1.7	8.8	6.20 (3.76 to 8.64)	41.3
Shoubridge 1985 * ‡ || †† ‡‡ ***	5	28.1 ± 3.4	5	20.0 ± 2.9	6.9	8.10 (4.18 to 12.02)	40.5
Williams 2009 * ‡ || ** ‡‡ ***	12	59.4 ± 9.4	12	56.4 ± 4.9	4.8	3.00 (-2.96 to 8.96)	25.1
Williams 2009 * ‡ || †† ‡‡ ***	12	24.9 ± 4.2	12	19.9 ± 6.2	6.5	5.00 (0.76 to 9.24)	5.3
**Total**	**130**		**131**				
**Chi-square test for heterogeneity = 62.29, P < 0.00001, I^2 = 78%**							
**μmol / min x gram protein**							
Ren 1993 * ‡ || †† ‡‡ ***	8	141.0 ± 22.6	8	101.0 ± 14.1	100.0	40.00 (21.51 to 58.49)	39.6

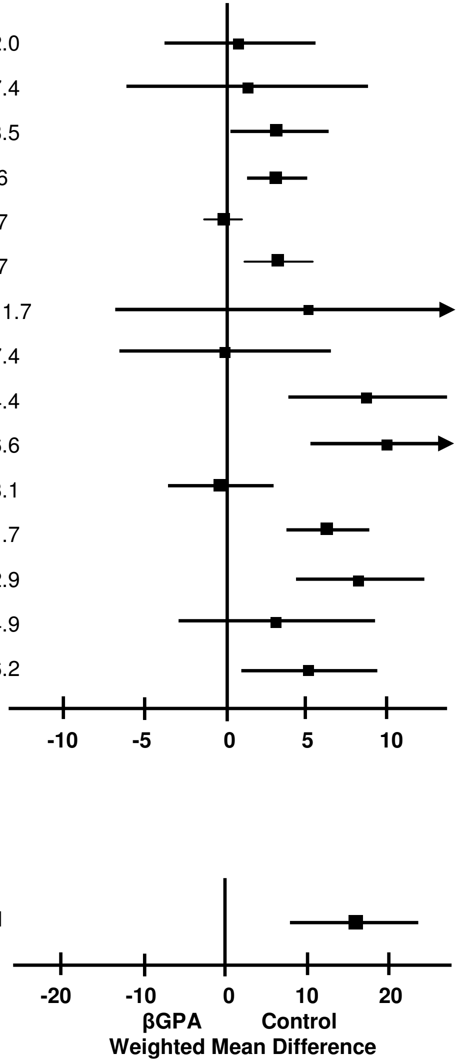

G

Study (Reference)	Intervention N	Intervention Mean Concentration ± SD	Control N	Control Mean Concentration ± SD	Weight, %	Weighted Mean Difference (95% CI)	Weighted Mean Percentage Change (%)
**μmol glucosyl equivalent / gram wet weight**							
Meyer 1986 * ‡ ¶ †† §§ ***	4	52.0 ± 8.0	4	32.0 ± 4.0	11.1	20.00 (11.23 to 28.77)	62.5
Ohira 1994 * ‡ || ** ‡‡ ***	6	51.1 ± 16.7	6	30.3 ± 9.8	5.1	20.80 (5.33 to 36.27)	68.6
Ohira 1994 † ‡ || †† ‡‡ ***	6	40.0 ± 4.4	6	14.0 ± 1.5	20.4	26.00 (22.28 to 29.72)	185.7
Ren 1993 * ‡ || †† ‡‡ ***	6	49.0 ± 5.4	6	24.4 ± 3.7	17.3	24.60 (19.38 to 29.82)	100.8
Shoubridge 1985 * ‡ || †† ‡‡ ***	10	76.0 ± 14.6	11	41.4 ± 4.8	10.1	34.60 (25.12 to 44.08)	83.6
Tanaka 1997 * ‡ || ** ‡‡ ***	6	50.3 ± 5.4	6	29.9 ± 6.1	14.7	20.40 (13.87 to 26.93)	71.7
Tanaka 1997 * ‡ || †† ‡‡ ***	6	70.2 ± 11.0	6	36.5 ± 6.1	9.4	33.70 (23.61 to 43.79)	92.3
Tanaka 1997 * ‡ || †† ‡‡ ***	6	69.8 ± 7.1	6	34.5 ± 7.4	11.9	35.30 (27.12 to 43.48)	102.3
**Total**	**50**		**51**		**100.0**	**26.70 (22.81 to 30.60)**	**96.2**

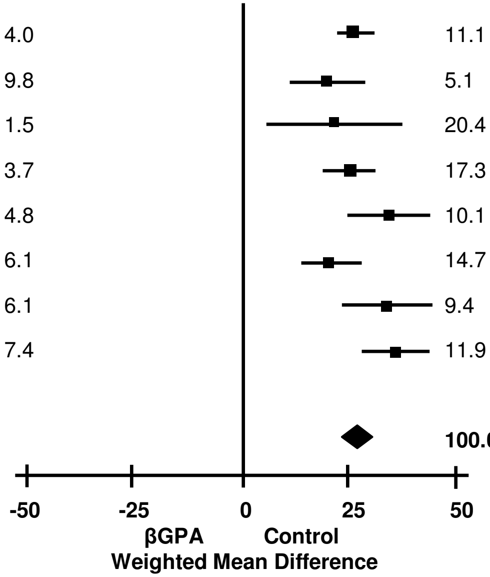

Chi-square test for heterogeneity = 15.69, P = 0.03, I^2 = 55%

Test for overall effect: Z = 13.43, P < 0.00001

H

Study (Reference)	Intervention N	Intervention Mean Concentration ± SD	Control N	Control Mean Concentration ± SD	Weight, %	Weighted Mean Difference (95% CI)	Weighted Mean Percentage Change (%)
**Non-diabetic rodents, mM, fasting**							
Mukhina 2008 * ‡ ¶ ** |||| ***	7	6.4 ± 0.2	7	7.9 ± 0.9	21.9	-1.48 (-2.12 to -0.84)	-29.0
Ohira 1994 * ‡ || ‡‡ ***	6	3.3 ± 0.7	6	3.8 ± 0.3	22.9	-0.47 (-1.09 to 0.15)	-13.2
Ohira 1994 † ‡ || ‡‡ ***	6	3.3 ± 0.4	6	3.9 ± 0.3	30.1	-0.58 (-1.00 to -0.16)	-15.4
Pandke 2008 * § || ‡‡ ***	12	8.3 ± 0.7	12	8.7 ± 0.7	25.1	-0.40 (-0.95 to 0.15)	-4.6
**Totaal**	**31**		**31**		**100.0**	**-0.71 (-1.14 to -0.27)**	**-13.9**
**Chi-square test for heterogeneity = 7.58, P = 0.06, I^2 = 60%**						**Test for overall effect: Z = 3.17; P = 0.002**	
**Non-diabetic rodents, mM, non-fasting**							
Moerland 1989 † ‡ || |||| ***	8	8.0 ± 1.7	8	7.2 ± 1.7	43.2	0.80 (-0.86 to 2.46)	11.1
Zong 2002 † ‡ ¶ ‡‡ ***	5	10.7 ± 0.5	5	12.1 ± 0.3	56.8	-1.40 (-1.88 to -0.92)	-11.6
**Total**	**13**		**13**				
**Chi-square test for heterogeneity = 6.25, P = 0.01, I^2 = 84%**							
**Diabetic rodents, mM, non-fasting**							
Meglasson 1993 † ‡ § ¶ ||| ¶¶	5	21.0 ± 1.1	5	31.0 ± 4.9	48.2	10.00 (-14.40 to -5.60)	-32.3
Meglasson 1993 † § ¶ ||| ¶¶	5	9.6 ± 2.7	5	18.1 ± 4.0	51.8	-8.50 (-12.74 to -4.26)	-47.0
**Total**	**10**		**10**		**100.0**	**-9.22 (-12.28 to -6.17)**	**-39.7**
**Chi-square test for heterogeneity = 0.23, P = 0.63, I^2 = 0%**						**Test for overall effect: Z = 5.92; P < 0.00001**	

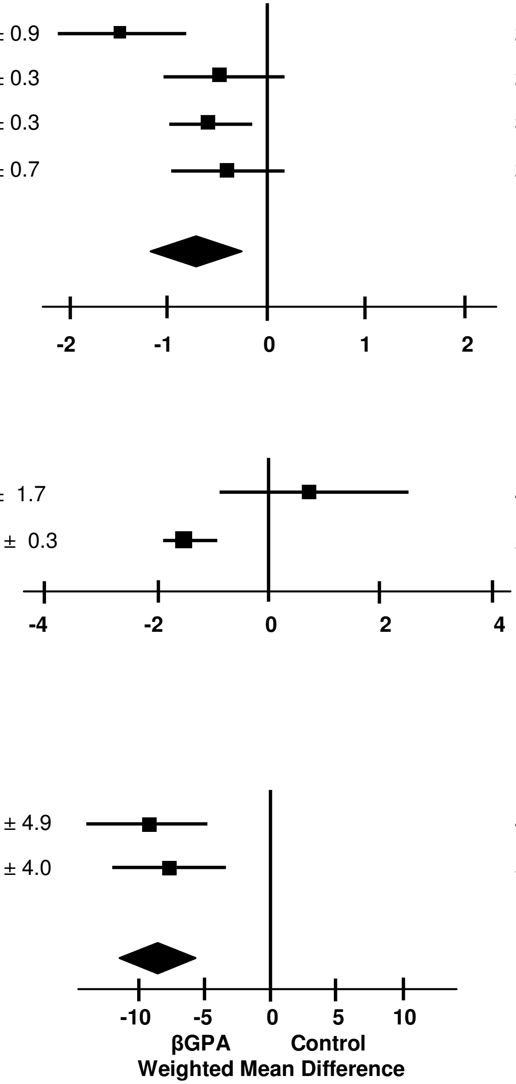

I

Study (Reference)	Intervention N	Intervention Mean Concentration ± SD	Control N	Control Mean Concentration ± SD	Weight, %	Weighted Mean Difference (95% CI)	Weighted Mean Percentage Change (%)
**Non-diabetic rodents, non-fasting, μU/ml**							
Zong 2002 † ‡ ¶ ‡‡ ***	5	31.0 ± 11.2	5	28.0 ± 13.4	100.0	3.00 (-12.31 to 18.31)	10.7
**Non-diabetic rodents, fasting, ng/ml**							
Pandke 2008 * § ‖ ‡‡ ***	12	1.5 ± 0.4	12	1.7 ± 0.4	100.0	-0.20 (-0.48 to 0.08)	-11.8
**Non-diabetic rodents, fasting, ng/ml**							
Ohira 1994 * ‡ ‖ ** ‡‡ ***	6	21.5 ± 12.5	6	36.6 ± 9.1	100.0	-15.10 (-27.45 to -2.75)	-41.3
**Diabetic rodents, fasting, nM**							
Meglasson 2003 (ob/ob mice)	6	1.4 ± 0.5	6	4.5 ± 1.0	100.0	-3.10 (-3.98 to -22.22)	-78.9

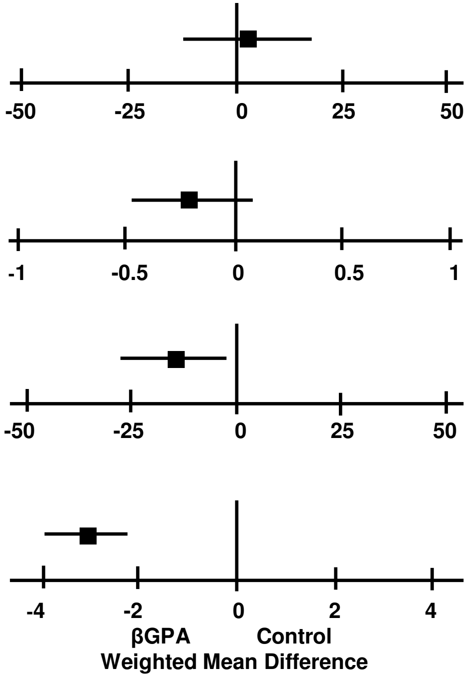

J

Study (Reference)	Intervention N	Intervention Mean Concentration ± SD	Control N	Control Mean Concentration ± SD	Weight, %	Weighted Mean Difference (95% CI)	Weighted Mean Percentage Change (%)
**Initial peak force (gram)**							
Fitch 1978 * ‡ ¶ ** ‡‡ ***	5	810.0 ± 470.0	4	1200.0 ± 170.0	8.4	-390.00 (-834.38 to 54.38)	-32.5
Fitch 1978 * ‡ ¶ †† ‡‡ ***	6	1200.0 ± 350.0	5	890.0 ± 310.0	9.9	310.00 (-80.21 to 700.21)	34.8
Meyer 1986 * ‡ ¶ †† §§ ***	4	825.0 ± 180.0	4	875.0 ± 160.0	16.2	-50.00 (-286.01 to 186.01)	-5.7
Meyer 1989 * ‡ ¶ †† §§ ***	3	958.0 ± 183.6	6	789.0 ± 205.8	14.8	169.00 (-96.08 to 434.08)	21.4
Petrovsky 1980 (1) * § || ** ‡‡ ***	6	752.0 ± 69.0	6	1065.0 ± 76.0	23.7	-313.00 (-395.14 to -230.86)	-29.4
Petrovsky 1980 (2) * § || †† ‡‡ ***	6	1200.0 ± 76.0	6	1350.0 ± 88.0	23.3	-150.00 (-243.04 to -56.96)	-11.1
Tullson 1996 * ‡ ¶ †† ‡‡ ***	5	2411.0 ± 234.8	3	2375.0 ± 640.9	3.7	36..00 (-717.82 to 789.82)	1.5
**Total**	**24**		**25**				

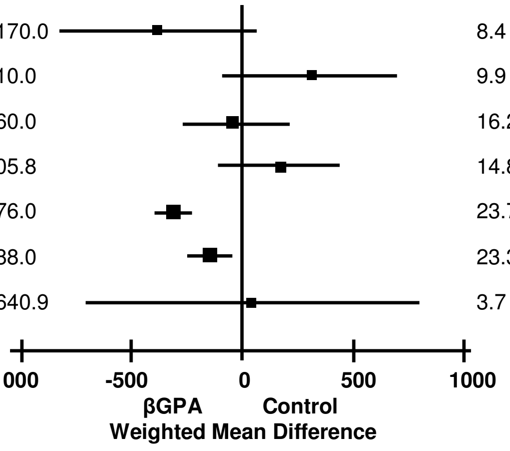

Chi-square test for heterogeneity = 25.02, P =0.0003, I^2 = 76%

K

Peak twitch force (gram)

Study (Reference)	Intervention N	Intervention Mean Concentration ± SD	Control N	Control Mean Concentration ± SD	Weight, %	Weighted Mean Difference (95% CI)	Weighted Mean Percentage Change (%)
Foley 1994 * ‡ ¶ †† ‡‡ ***	5	38.0 ± 4.5	5	38.0 ± 8.9	12.8	0.00 (-8.76 to 8.76)	0.0
Mainwood 1982-2 * ‡ || †† ‡‡ ***	6	21.9 ± 5.9	6	18.4 ± 3.7	16.1	3.50 (-2.05 to 9.05)	19.0
Nasledov 1996 * ‡ ¶ ** |||| *** ¶ ¶	6	14.0 ± 1.6	6	11.0 ± 2.0	19.0	3.00 (0.95 to 5.05)	27.3
Nasledov 1996 * ‡ ¶ ** |||| *** ¶ ¶	5	28.0 ± 5.0	5	25.0 ± 2.8	16.7	3.00 (-2.02 to 8.02)	12.0
Wakatsuki 1994 * ‡ || ** ‡‡ ***	6	12.7 ± 2.7	6	17.8 ± 6.1	16.3	-5.10 (-10.45 to 0.25)	-28.7
Wakatsuki 1995 * ‡ || †† ‡‡ ***	6	12.5 ± 1.2	6	20.2 ± 2.0	19.1	-7.70 (-9.55 to -5.85)	-38.1
**Total**	**34**		**34**				

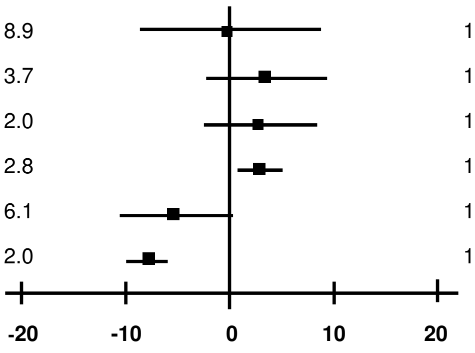

Chi-square test for heterogeneity = 68.16, P < 0.00001, I^2 = 93%

Peak twitch force (N/cm²)

Study (Reference)	Intervention N	Intervention Mean Concentration ± SD	Control N	Control Mean Concentration ± SD	Weight, %	Weighted Mean Difference (95% CI)	Weighted Mean Percentage Change (%)
Eijnde 2004 † ‡ ¶ ** ‡‡ ¶¶	12	5.1 ± 1.4	10	4.3 ± 1.3	18.4	0.80 (-0.31 to 1.91)	18.6
Eijnde 2004 † ‡ ¶ †† ‡‡ ¶¶	12	7.9 ± 1.9	11	9.4 ± 3.1	13.3	-1.48 (-3.63 to 0.67)	-16.0
Gagnon 2002 * ‡ ¶ ** |||| ¶¶	6	0.6 ± 0.5	6	1.0 ± 0.8	20.1	-0.40 (-1.10 to 0.30)	-40.0
Gagnon 2002 * ‡ ¶ ‡‡ |||| ¶¶	6	1.4 ± 0.9	5	0.9 ± 0.5	19.5	0.56 (-0.30 to 1.42)	55.6
Levine 1996 * ‡ ¶ †† §§ ††	15	3.7 ± 1.22	15	6.4 ± 1.3	19.3	-2.68 (-3.58 to -1.78)	-42.2
Mainwood 1982-2 * ‡ || †† ‡‡ ***	6	9.7 ± 2.9	6	7.4 ± 2.5	9.5	2.30 (-0.76 to 5.36)	31.1
**Total**	**57**		**53**				

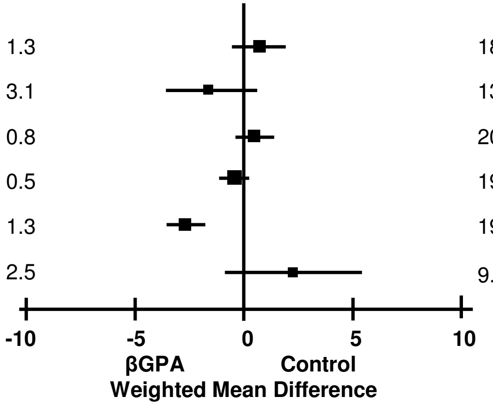

Chi-square test for heterogeneity = 37.85, P < 0.00001, I^2 = 87%

L

Study (Reference)	Intervention N	Intervention Mean Concentration ± SD	Control N	Control Mean Concentration ± SD	Weight, %	Weighted Mean Difference (95% CI)	Weighted Mean Percentage Change (%)
**Peak tetanic force (gram)**							
Foley 1994 * ¶ †† ‡‡ ***	5	130.0 ± 26.8	5	167.0 ± 47.0	3.5	-37.00 (-84.41 to 10.41)	-22.2
Mainwood 1982 * ‡ ‖ †† ‡‡ ***	23	89.9 ± 17.8	23	97.8 ± 19.7	37.9	-7.90 (-18.72 to 2.92)	-8.1
Mainwood 1982-2 * ‡ ‖ †† ‡‡ ***	6	78.5 ± 13.5	6	85.0 ± 12.0	26.7	-6.50 (-20.93 to 7.93)	-7.6
Wakatsuki 1994 * ‡ ‖ ** ‡‡ ***	6	55.4 ± 11.5	6	75.0 ± 22.3	16.3	-19.60 (-39.67 to 0.47)	-26.1
Wakatsuki 1995 * ‡ ‖ †† ‡‡ ***	6	53.1 ± 13.0	6	81.9 ± 22.3	15.6	-28.80 (-49.44 to -8.16)	-35.2
**Total**	**46**		**46**		**100.0**	**-13.71 (-22.75 to -4.67)**	**-15.5**

Chi-square test for heterogeneity = 5.26, P = 0.26, I^2 = 24%

Test for overall effect: Z = 2.97; P = 0.003

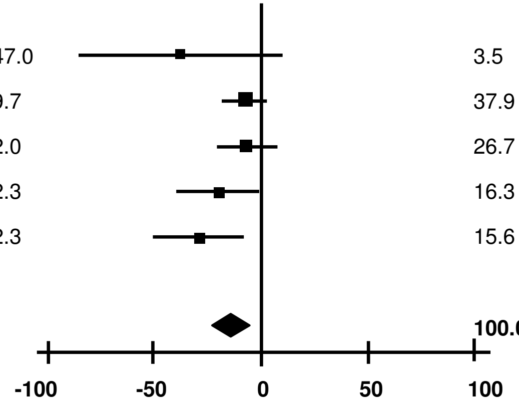

Study (Reference)	Intervention N	Intervention Mean Concentration ± SD	Control N	Control Mean Concentration ± SD	Weight, %	Weighted Mean Difference (95% CI)	Weighted Mean Percentage Change (%)
**Peak tetanic force (N/cm^2)**							
Eijnde 2004 (1) † ‡ ¶ ** ‡‡ ¶¶	12	19.9 ± 5.1	10	19.5 ± 4.4	14.2	0.38 (-3.60 to 4.36)	2.1
Eijnde 2004 (2) † ‡ ¶ †† ‡‡ ¶¶	12	24.0 ± 6.8	11	27.1 ± 5.7	13.1	-3.04 (-8.14 to 2.06)	-11.4
Gagnon 2002 * ‡ ¶ ** ‖‖ ¶¶	6	6.6 ± 5.1	6	9.3 ± 6.6	11.5	-2.70 (-9.37 to 3.97)	-29.0
Gagnon 2002 * ‡ ¶ ‡‡ ‖‖ ¶¶	6	9.9 ± 5.8	5	6.1 ± 3.6	12.6	3.77 (-1.82 to 9.36)	62.3
Levine 1996 * ‡ ¶ †† §§ ††	15	8.7 ± 1.6	15	14.6 ± 2.6	15.9	-5.90 (-7.45 to -4.35)	-40.4
Mainwood 1982-2 * ‡ ‖ †† ‡‡ ***	6	34.7 ± 7.1	6	34.3 ± 12.3	7.3	0.40 (-10.93 to 11.73)	1.2
Moerland 1994 (1) † ‡ ‖ ** ‖‖ ***	9	31.7 ± 3.3	6	25.1 ± 3.7	14.5	6.60 (2.96 to 10.24)	26.3
Moerland 1994 (2) † ‡ ‖ †† ‖‖ ***	8	27.9 ± 9.9	9	31.7 ± 3.3	10.9	-3.80 (-10.98 to 3.38)	-12.0
**Total**	**74**		**68**				

Chi-square test for heterogeneity = 48.86, P < 0.00001, I^2 = 86%

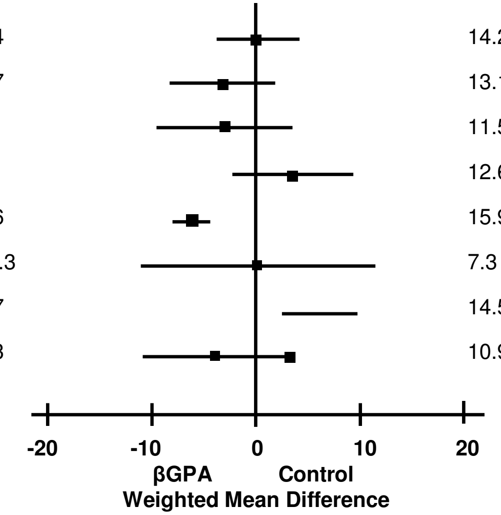

M

Study (Reference)	Intervention N	Intervention Mean Concentration ± SD	Control N	Control Mean Concentration ± SD	Weight, %	Weighted Mean Difference (95% CI)	Weighted Mean Percentage Change (%)
De Tata 1993 (1) * ‡ ** ‡‡ †††	5	152.0 ± 6.6	5	192.0 ± 22.6	5.4	-40.00 (-60.65 to -19.35)	-16.9
De Tata 1993 (2) * ‡ †† ‡‡ †††	5	128.0 ± 19.0	5	154.0 ± 13.9	5.4	-26.00 (-46.67 to -5.33)	-20.8
Freyssenet 1994 * ‡ ¶ †† ‡‡ ***	8	117.6 ± 7.8	8	132.4 ± 11.4	5.9	-14.80 (-24.37 to -5.23)	-11.2
Freyssenet 1995 (1) * ‡ ¶ ** ‡‡ ***	7	105.4 ± 10.4	8	116.7 ± 12.7	5.9	-11.30 (-23.00 to 0.40)	-9.7
Freyssenet 1995 (2) * ‡ ¶ †† ‡‡ ***	7	117.6 ± 7.8	8	134.5 ± 12.1	5.9	-16.90 (-27.08 to -6.72)	-12.6
Gori 1988 (1) * ‡ || ** ‡‡ ***	10	76.0 ± 7.6	10	101.0 ± 21.2	5.8	-25.00 (-38.95 to -11.05)	-24.8
Gori 1988 (2) * ‡ || †† ‡‡ ***	10	96.0 ± 15.8	10	119.0 ± 19.9	5.7	-23.00 (-38.76 to -7.24)	-19.3
Lai 1990 (1) * § ¶ ** ‡‡ ***	6	239.0 ± 24.0	6	255.0 ± 26.0	4.9	-16.00 (-44.31 to 12.31)	-6.3
Lai 1990 (2) * § ¶ †† ‡‡ ***	6	2490.0 ± 230.0	6	3050.0 ± 150.0	0.4	-560.00 (-779.71 to -340.29)	-18.4
Lai 1990 (3) * § ¶ †† ‡‡ ***	6	513.0 ± 34.0	6	628.0 ± 39.0	4.0	-115.00 (-156.40 to -73.60)	-18.3
Levine 1996 * ‡ ¶ †† §§ †††	16	446.0 ± 36.0	16	646.0 ± 36.0	5.1	-200.00 (-224.95 to -175.05)	-31.0
Mukhina 2008 * ‡ ¶ ** |||| ***	7	88.3 ± 4.8	7	89.0 ± 5.5	6.1	-0.67 (-6.08 to 4.74)	-0.8
Ohira 2003 * ‡ || ** ‡‡ ***	5	114.0 ± 6.7	5	149.0 ± 13.4	5.8	-35.00 (-48.15 to -21.85)	-23.5
Ohira 2003 * ‡ || †† ‡‡ ***	5	127.0 ± 11.2	5	164.0 ± 4.5	5.9	-37.00 (-47.55 to -26.45)	-22.6
Petrovsky 1980 * § || ** ‡‡ ***	6	118.0 ± 12.0	6	126.0 ± 18.0	5.6	-8.00 (-25.31 to 9.31)	-6.3
Petrovsky 1980 * § || †† ‡‡ ***	6	184.0 ± 26.0	6	264.0 ± 28.0	4.7	-80.00 (-110.57 to -49.43)	-30.3
Tanaka 1997 (1) * ‡ || ** ‡‡ ***	12	84.0 ± 13.9	12	126.0 ± 10.4	5.9	-42.00 (-51.80 to 32.20)	-33.3
Wakatsuki 1994 * ‡ || ** ‡‡ ***	6	113.0 ± 4.9	6	149.0 ± 17.2	5.7	-36.00 (-50.27 to -21.73)	-24.2
Wakatsuki 1995 * ‡ || †† ‡‡ ***	6	127.0 ± 12.3	6	164.0 ± 7.4	5.9	-37.00 (-48.43 to -25.57)	-22.6
**Total**	**139**		**141**		**100.0**	**-41.84 (-55.87 to -27.81)**	**-19.8**

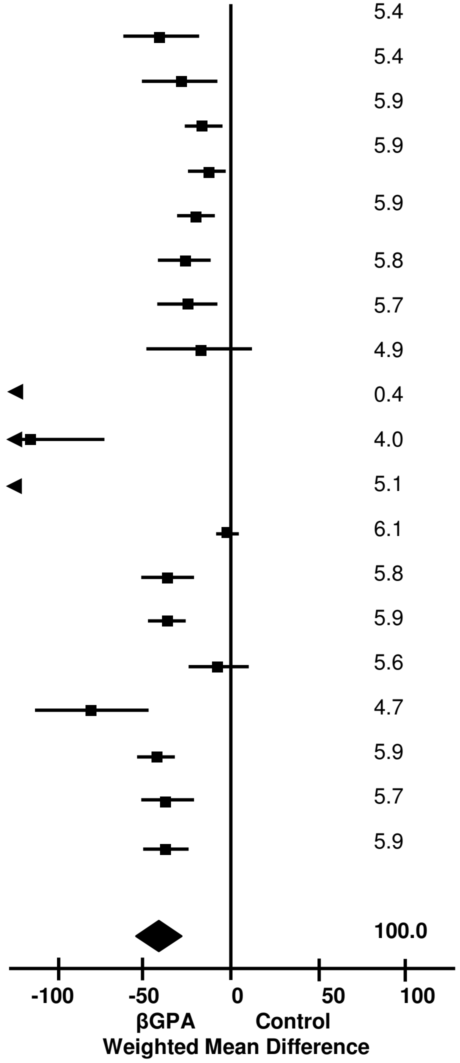

Chi-square test for heterogeneity = 361.41, P < 0.00001, I^2 = 95%

Test for overall effect: Z = 5.84; P < 0.00001
