# Supplementary material for: The Effect of the Creatine Analogue Beta-guanidinopropionic Acid on Energy Metabolism: A Systematic Review
Source: PLoS One. 2013 Jan 9;8(1):e52879. doi: 10.1371/journal.pone.0052879 (PMC3541392; doi:10.1371/journal.pone.0052879)

A

CK-MM, percentage of total

Study (Reference)	Intervention N	Intervention Mean Concentration ± SD	Control N	Control Mean Concentration ± SD	Weight, %	Weighted Mean Difference (95% CI)	Weighted Mean Percentage Change (%)
Boehm 2003 * ‡ ¶ ‡‡ ||||	4	25.3 ± 0.1	4	28.1 ± 3.3	32.6	-2.80 (-6.04 to 0.44)	-10.0
Field 1991 * ‡ || ** ||||	5	66.2 ± 7.4	5	60.9 ± 4.7	13.7	5.30 (-2.37 to 12.97)	8.7
Field 1991 (2) * ‡ ¶ ** ||||	5	60.1 ± 3.4	5	60.8 ± 3.6	26.5	-0.70 (-5.00 to 3.60)	-1.2
Pelouch 1996 * ‡ || ** ||||	6	62.6 ± 3.9	6	59.6 ± 3.5	27.2	3.00 (-1.18 to 7.18)	5.0
**Total**	**20**		**20**				

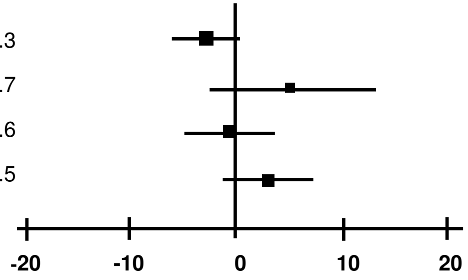

Chi-square test for heterogeneity = 6.76; P = 0.08; I² = 56%

CK-MB, percentage of total

Study (Reference)	Intervention N	Intervention Mean Concentration ± SD	Control N	Control Mean Concentration ± SD	Weight, %	Weighted Mean Difference (95% CI)	Weighted Mean Percentage Change (%)
Boehm 2003 * ‡ ¶ ‡‡ ||||	4	21.5 ± 2.1	4	18.6 ± 3.3	7.9	2.90 (-0.93 to 6.73)	15.6
Field 1991 * ‡ || ** ||||	5	22.6 ± 5.6	5	24.7 ± 2.7	4.0	-2.10 (-7.53 to 3.33)	-8.5
Field 1991 (2) * ‡ ¶ ** ||||	5	20.2 ± 4.0	5	20.7 ± 0.9	8.9	-0.50 (-4.11 to 3.11)	-2.4
Pelouch 1996 * ‡ || ** ||||	6	12.5 ± 1.1	6	11.2 ± 1.0	79.2	1.30 (0.13 to 2.47)	11.6
**Total**	**20**		**20**				

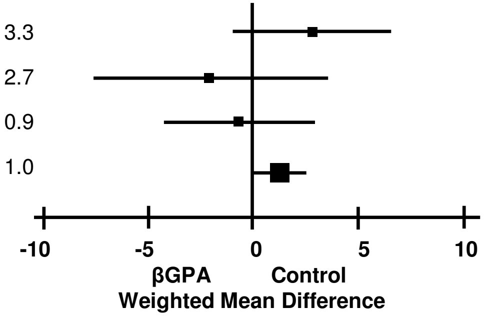

Chi-square test for heterogeneity = 3.04; P = 0.39; I² = 1%

B

Study (Reference)	Intervention N	Intervention Mean Concentration ± SD	Control N	Control Mean Concentration ± SD	Weight, %	Weighted Mean Difference (95% CI)	Weighted Mean Percentage Change (%)
**CK-BB, percentage of total**							
Boehm 2003 * ‡ ¶ ‡‡ ||||	4	5.0 ± 0.2	4	3.5 ± 1.3	20.4	1.50 (0.21 to 2.79)	42.9
Field 1991 * ‡ || ** ||||	5	4.5 ± 1.6	5	5.5 ± 1.3	12.8	-1.00 (-2.81 to 0.81)	-18.2
Field 1991 (2) * ‡ ¶ ** ||||	5	3.9 ± 1.1	5	3.6 ± 0.7	23.5	0.30 (-0.84 to 1.44)	8.3
Pelouch 1996 * ‡ || ** ||||	6	2.7 ± 0.6	6	2.6 ± 0.02	43.2	0.10 (-0.39 to 0.59)	3.8
**Total**	**20**		**20**				

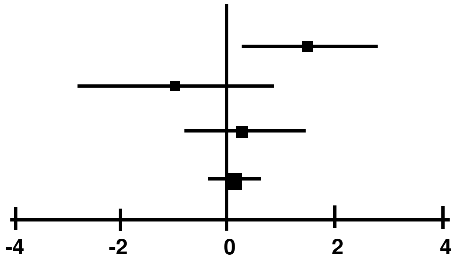

Chi-square test for heterogeneity = 5.79; P = 0.12; I² = 48%

Study (Reference)	Intervention N	Intervention Mean Concentration ± SD	Control N	Control Mean Concentration ± SD	Weight, %	Weighted Mean Difference (95% CI)	Weighted Mean Percentage Change (%)
**CK-Mi, percentage of total**							
Boehm 2003 * ‡ ¶ ‡‡ ||||	4	25.3 ± 0.1	4	28.1 ± 3.3	38.9	-2.80 (-6.04 to 0.44)	38.9
Field 1991 * ‡ || ** ||||	5	6..8 ± 1.6	5	8.9 ± 4.7	21.6	-2.10 (-6.44 to 2.24)	21.6
Field 1991 (2) * ‡ ¶ ** ||||	5	15.2 ± 4.7	5	14.8 ± 3.1	16.6	0.40 (-4.55 to 5.35)	16.6
Pelouch 1996 * ‡ || ** ||||	6	21.3 ± 4.1	6	26.5 ± 3.3	22.8	-5.20 (-9.43 to -0.97)	22.8
**Total**	**20**		**20**				

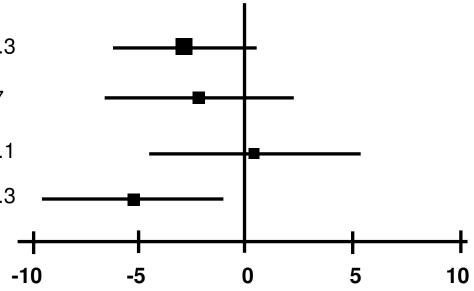

Chi-square test for heterogeneity = 2.93; P = 0.40; I² = 0%

Study (Reference)	Intervention N	Intervention Mean Concentration ± SD	Control N	Control Mean Concentration ± SD	Weight, %	Weighted Mean Difference (95% CI)	Weighted Mean Percentage Change (%)
**CK-Mi, mU/mg protein**							
O'Gorman 1997 * ‡ || ‡‡ ¶¶	4	112.0 ± 25.0	4	148.0 ± 31.0	100.0	-36.00 (-75.03 to 3.03)	-24.3

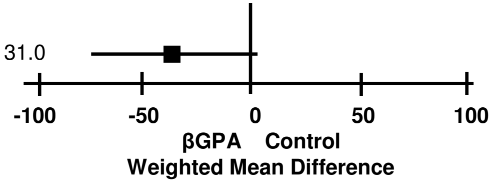

C

Study (Reference)	Intervention N	Intervention Mean Concentration ± SD	Control N	Control Mean Concentration ± SD	Weight, %	Weighted Mean Difference (95% CI)	Weighted Mean Percentage Change (%)
**Citrate synthase, μmol/min per g wet weight**							
Field 1991 * ‡ ǁ ** ǁǁ	5	55.3 ± 10.6	5	60.6 ± 9.5	24.9	-5.30 (-17.80 to 7.20)	-8.7
Field 1991 (2) * ‡ ¶ ** ǁǁ	5	62.3 ± 7.5	5	62.3 ± 4.4	66.3	0.00 (-7.67 to 7.67)	0.0
Shoubridge 1985 * ‡ ǁ ** ǁǁ	5	98.7 ± 20.3	5	111.0 ± 12.9	8.8	-12.30 (-33.38 to 8.78)	-11.1
**Total**	**15**		**15**		**100.0**	**-2.40 (-8.64 to 3.84)**	**-6.6**
**Chi-square test for heterogeneity = 1.43; P = 0.49; I² = 0%**						**Test for overall effect: Z = 0.75; P = 0.45**	
**Hexokinase, μmol/g wet weight**							
Shoubridge 1985 * ‡ ǁ ** ǁǁ	4	5.8 ± 0.7	4	6.1 ± 0.1	100.0	-0.27 (-0.96 to 0.42)	-4.9
**Hexokinase, U/g wet weight**							
O'Gorman 1997 * ‡ ǁ ‡‡ ¶¶	5	1.4 ± 0.4	5	3.0 ± 0.3	100.0	-1.60 (-2.04 to -1.16)	-53.3

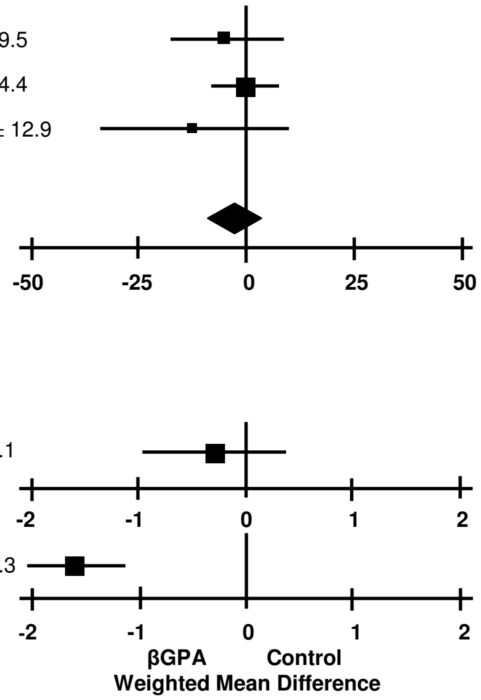

D

Study (Reference)	Intervention N	Intervention Mean Concentration ± SD	Control N	Control Mean Concentration ± SD	Weight, %	Weighted Mean Difference (95% CI)	Weighted Mean Percentage Change (%)
**Baseline left ventricular systolic pressure, mm Hg**							
Neubauer 1999 * ‡ ¶ ** ||||	10	138.0 ± 12.7	10	134.0 ± 6.3	100.0	4.00 (-4.77 to 12.77)	3.0
**Baseline left ventricular fractional shortening, percentage**							
Lindblom 2008 † ‡ ¶ ‡‡ ||||	15	27.0 ± 3.9	10	55.0 ± 6.3	50.6	-28.00 (-32.38 to 23.62)	-50.9
Lorentzon 2007 * ‡ || ‡‡ ||||	25	34.0 ± 9.5	23	45.0 ± 10.6	49.4	-11.00 (-16.70 to -5.30)	-24.6
**Total**	**40**		**33**		**100.0**	**-19.60 (-36.26 to -2.94)**	**-33.61**
**Chi-square test for heterogeneity = 21.48; P < 0.00001; I^2 = 95%**						**Test for overall effect: Z = 2.31; P = 0.02**	
**Baseline cardiac output, mL/min**							
Neubauer 1999 * ‡ ¶ ** ||||	10	144.0 ± 12.7	10	125.0 ± 28.5	100.0	19.00 (-0.30 to 38.30)	15.2
**Left ventricular end-diastolic pressure, mm Hg**							
Neubauer 1999 * ‡ ¶ ** ||||	10	2.9 ± 2.2	10	9.4 ± 2.5	100.0	-6.50 (-8.58 to -4.42)	-69.1
**Peak left ventricular developed pressure during aortic occlusion, mmHg**							
Neubauer 1999 * ‡ ¶ ** ||||	10	210.0 ± 12.7	10	232.0 ± 19.0	100.0	-22.00 (-36.13 to -7.87)	-9.5
**Left ventricular fractional shortening during stress induced by pacing, percentage**							
Lorentzon 2007 * ‡ || ‡‡ ||||	25	43.0 ± 10.0	23	51.0 ± 4.8	100.0	-8.00 (-12.38 to 3.62)	-15.7

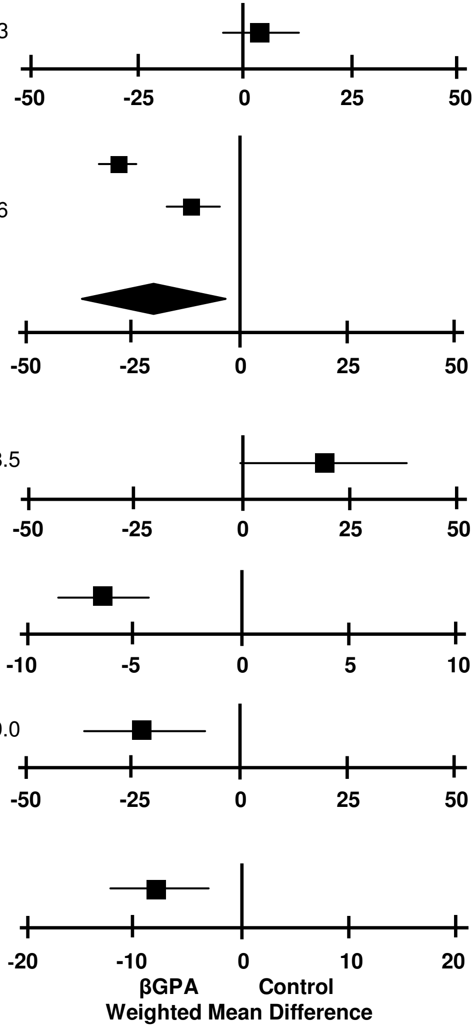

E

Study (Reference)	Intervention N	Intervention Mean Concentration ± SD	Control N	Control Mean Concentration ± SD	Weight, %	Weighted Mean Difference (95% CI)	Weighted Mean Percentage Change (%)
**Left Ventricular Developed Pressure, mm Hg**							
Gorman 1997	5	77.0 ± 11.2	5	142.0 ± 22.4	14.5	-65.00 (-86.91 to -43.09)	-45..8
Kapelko 1988 * ‡ ** ||||	4	83.3 ± 7.2	5	93.2 ± 6.5	15.2	-9.90 (-18.97 to -0.83)	-11.6
Mekhfi 1990 * ‡ || ** ¶¶	6	98.0 ± 53.7	6	167.0 ± 22.4	11.9	-69.00 (-119.96 to -18.04)	-41.3
Neubauer 1998 * ‡ ¶ ** ||||	6	60.0 ± 17.1	6	94.0 ± 14.7	14.8	-34.00 (-52.04 to -15.96)	-36.2
Neubauer 1999 * ‡ ¶ ** ||||	6	58.0 ± 17.1	6	87.0 ± 41.7	13.4	-29.00 (-64.99 to 6.99)	-33.3
Shoubridge 1985 * ‡ || ** ||||	5	34.0 ± 10.0	5	46.0 ± 9.0	15.1	-12.00 (-23.79 to -0.21)	-26.1
Zweier 1991 * ‡ ¶ ** ||||	14	141.0 ± 12.0	14	240.0 ± 8.0	15.2	-99.00 (-106.55 to -91.45)	-41.2
**Total**	**45**		**46**		**100.0**	**-44.90 (-81.81 to -7.99)**	**-35.6**
**Chi-square test for heterogeneity = 282.82; P< 0.00001; I² = 98%**						**Test for overall effect: Z = 2.38; P = 0.02**	
**Cardiac output, ml/min**							
Kapelko 1988 * ‡ ** ||||	4	32.0 ± 6.0	5	35.0 ± 4.5	100.0	-3.00 (-10.07 to 4.07)	-8.6
**Cardiac output, ml/min per gram dry weight**							
Shoubridge 1985 * ‡ || ** ||||	5	456.0 ± 40.0	5	422.0 ± 36.0	100.0	34.00 (-13.17 to 81.17)	8.1

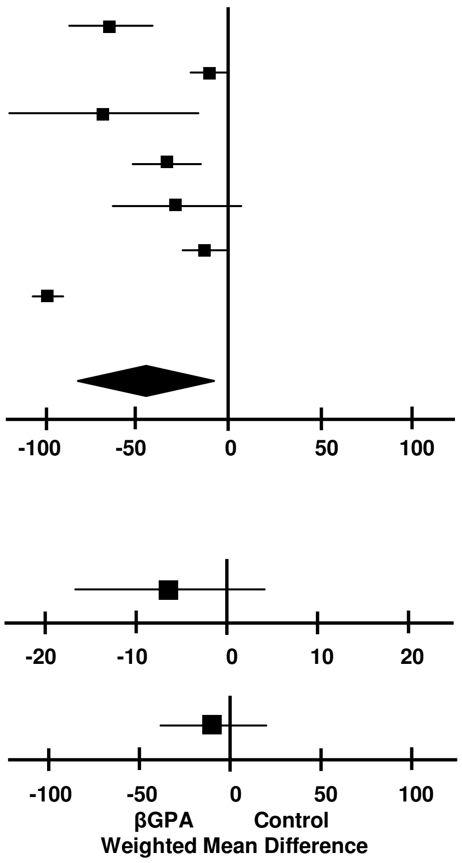

F

Study (Reference)	Intervention N	Intervention Mean Concentration ± SD	Control N	Control Mean Concentration ± SD	Weight, %	Weighted Mean Difference (95% CI)	Weighted Mean Percentage Change (%)
**Left Ventricular Developed Pressure, mm Hg**							
Kapelko 1988 * ‡ ** ||||	6	138.0 ± 19.6	13	161.0 ± 10.8	42.5	-23.00 (-39.75 to -6.25)	-14.3
Shoubridge 1985 * ‡ || ** ||||	5	29.0 ± 7.0	5	32.0 ± 4.0	57.5	-3.00 (-10.07 to 4.07)	-9.4
**Total**	**11**		**18**		**100.0**	**-11.50 (-30.88 to 7.88)**	**-12.6**
**Chi-square test for heterogeneity = 4.65; P = 0.03; I^2 = 78%**						**Test for overall effect: Z = 1.16; P = 0.03**	
**Cardiac output, mL/min**							
Kapelko 1988 * ‡ ** ||||	6	25.0 ± 12.3	13	31.0 ± 7.2	100.0	-6.00 (-16.56 to 4.56)	-19.4
**Cardiac output, mL/min per gram dry weight**							
Shoubridge 1985 * ‡ || ** ||||	5	379.0 ± 21.0	5	388.0 ± 25.0	100.0	-9.00 (-37.62 to 19.62)	-2.3

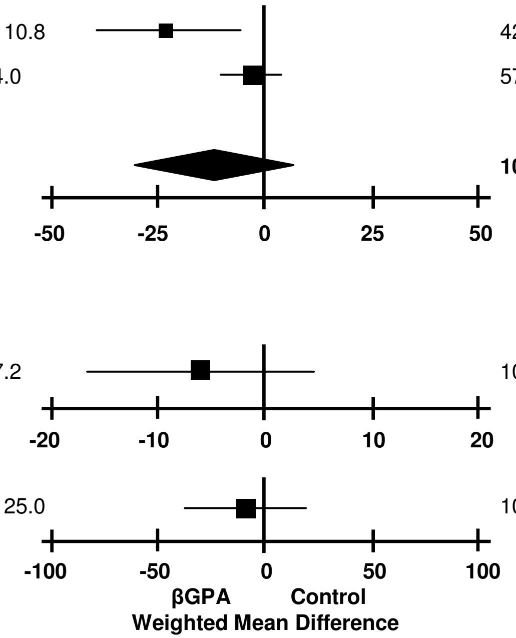

G

Study (Reference)	Intervention N	Intervention Mean Concentration ± SD	Control N	Control Mean Concentration ± SD	Weight, %	Weighted Mean Difference (95% CI)	Weighted Mean Percentage Change (%)
**Wet Heart Weight, grams**							
Boehm 2003 * ‡ ¶ ‡‡ ||||	9	1.33 ± 0.12	20	1.17 ± 0.49	8.7	0.16 (-0.07 to 0.39)	13.7
Clark 1994 * ‡ || ** ||||	5	1.35 ± 0.42	4	1.15 ± 0.08	3.2	0.20 (-0.18 to 0.58)	17.4
Horn 2001 * ‡ ¶ ** ||||	5	1.80 ± 1.79	7	1.81 ± 0.16	0.2	-0.01 (-1.58 to 1.56)	-0.6
Pelouch 1996 * ‡ || ** ||||	16	0.77 ± 0.12	8	0.78 ± 0.06	87.9	-0.01 (-0.08 to 0.06)	1.3
**Total**	**35**		**39**		**100.0**	**0.02 (-0.06 to 0.09)**	**7.0**
**Chi-square test for heterogeneity**	**= 2.83; P = 0.42; I² = 0%**					**Test for overall effect: Z = 0.39; P = 0.70**	
**Heart Weight to Body Weight Ratio**							
Boehm 2003 * ‡ ¶ ‡‡ ||||	9	3.9 ± 0.2	20	3.3 ± 0.9	19.7	0.58 (0.18 to 0.98)	18.2
Clark 1994 * ‡ || ** ||||	5	3.8 ± 0.2	4	2.9 ± 0.01	23.7	0.86 (0.67 to 1.05)	31.0
Horn 2001 * ‡ ¶ ** ||||	5	3.5 ± 0.3	7	3.6 ± 0.5	18.5	-0.13 (-0.59 to 0.33)	-2.8
Neubauer 1999 * ‡ ¶ ** ||||	6	3.5 ± 0.3	6	3.6 ± 0.6	16.4	-0.14 (-0.70 to 0.42)	-2.8
Pelouch 1996 * ‡ || ** ||||	16	3.8 ± 0.5	8	3.3 ± 0.3	21.7	0.51 (0.20 to 0.82)	15.2
**Total**	**35**		**39**				
**Chi-square test for heterogeneity**	**= 23.74; P < 0.0001; I² = 83%**						

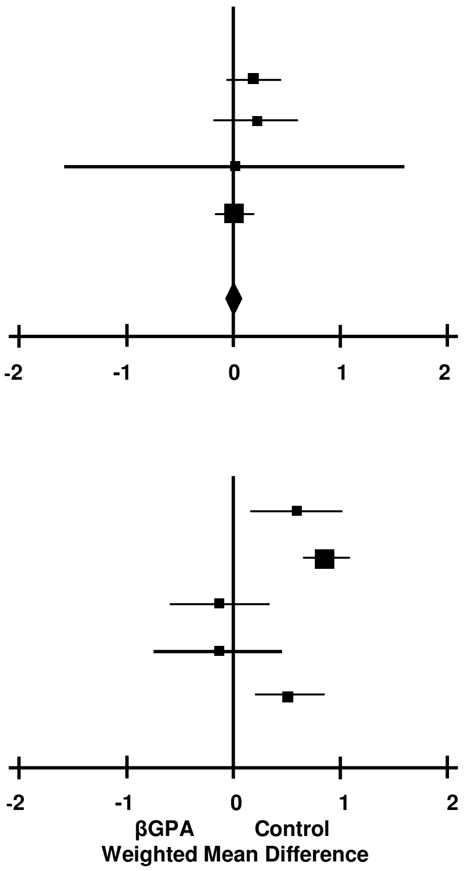

H

Study (Reference)	Intervention N	Intervention Mean Concentration ± SD	Control N	Control Mean Concentration ± SD	Weight, %	Weighted Mean Difference (95% CI)	Weighted Mean Percentage Change (%)
**Left Ventricle Weight, grams**							
Lorentzon 2007 * ‡ ‖ ‡‡ ‖‖	15	1.25 ± 0.04	15	0.93 ± 0.23	23.5	0.32 (0.20 to 0.44)	34.4
Mekhfi 1990 * ‡ ‖ ** ¶¶	12	1.01 ± 012	12	0.80 ± 0.12	24.8	0.21 (0.11 to 0.31)	26.3
Neubauer 1999 * ‡ ¶ ** ‖‖	6	0.92 ± 0.05	6	0.91 ± 0.10	25.1	0.01 (-0.08 to 0.10)	1.1
Pelouch 1996 * ‡ ‖ ** ‖‖	16	0.42 ± 0.09	8	0.43 ± 0.05	26.6	-0.02 (-0.07 to 0.04)	-2.3
**Total**	**49**		**41**				
**Chi-square test for heterogeneity = 36.11; P < 0.00001; I^2 = 92%**							
**Left Ventricle Weight to Body Weight Ratio**							
Adams 1995 * § ‖ ** ‖‖	8	2.2 ± 0.2	8	2.2 ± 0.2	26.7	0.00 (-0.15 to 0.15)	0.0
Lorentzon 2007 * ‡ ‖ ‡‡ ‖‖	15	2.8 ± 0.2	15	2.1 ± 0.6	23.1	0.70 (0.39 to 1.01)	33.3
Mekhfi 1990 * ‡ ‖ ** ¶¶	12	2.7 ± 0.2	12	2.1 ± 0.2	25.3	0.62 (0.40 to 0.84)	28.6
Pelouch 1996 * ‡ ‖ ** ‖‖	16	2.0 ± 0.4	8	1.9 ± 0.2	24.9	0.15 (-0.09 to 0.39)	5.3
**Total**	**45**		**39**		**100.0**	**0.36 (0.01 to 0.71)**	**19.3**
**Chi-square test for heterogeneity = 29.57; P < 0.00001; I^2 = 90%**						**Test for overall effect: Z = 2.00; P = 0.05**	

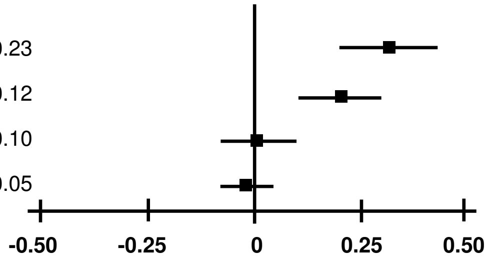

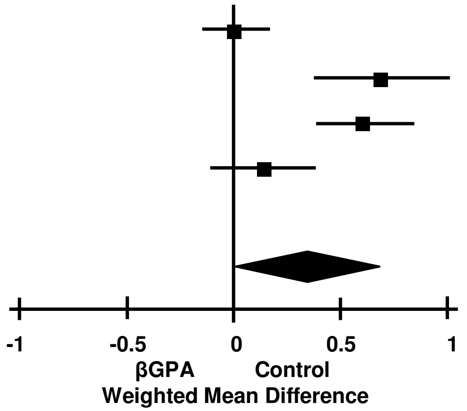

Supplement: Figure S2 — The effect of βGPA on the creatine kinase system of the heart. The effect of βGPA on myocardial total creatine (A), phosphocreatine (B), ATP, and creatine kinase activity (D). This is a random-effects model. Squares are weighted mean differences in body weight. The size of the squares represents study weight and horizontal lines represent 95% CIs. Arrowheads depict data outside the scale. Black diamonds are pooled estimates. The weighted mean percentage change was calculated from the percentage change per study and the sample size. * Rat; † Mouse; ‡ Male sex; § Female sex; ∥ Animal age ≤6 weeks; ¶ Animal age >6 weeks; ** Dose of βGPA = 1% in the diet or drinking water; †† Dose of βGPA = 2% in the diet or drinking water; ‡‡ Dose of βGPA = other; §§ Duration of intervention ≤3 weeks; ∥∥ Duration of intervention 3 to 10 weeks; ¶¶ Duration of intervention >10 weeks. (PDF) [file pone.0052879.s002.pdf]
